# Prediction of PD-L1 Expression in Neuroblastoma via Computational Modeling

**DOI:** 10.3390/brainsci9090221

**Published:** 2019-08-31

**Authors:** Salvo Danilo Lombardo, Mario Presti, Katia Mangano, Maria Cristina Petralia, Maria Sofia Basile, Massimo Libra, Saverio Candido, Paolo Fagone, Emanuela Mazzon, Ferdinando Nicoletti, Alessia Bramanti

**Affiliations:** 1 Department of Biomedical and Biotechnological Sciences, University of Catania, 95123 Catania, Italy; 2 IRCCS (Istituti di Ricovero e Cura a Carattere Scientifico) Centro Neurolesi Bonino Pulejo, C.da Casazza, 98124 Messina, Italy

**Keywords:** Neuroblastoma, PD-L1, computational modelling, Immunotherapy, COPASI

## Abstract

Immunotherapy is a promising new therapeutic approach for neuroblastoma (NBM): an anti-GD2 vaccine combined with orally administered soluble beta-glucan is undergoing a phase II clinical trial and nivolumab and ipilimumab are being tested in recurrent and refractory tumors. Unfortunately, predictive biomarkers of response to immunotherapy are currently not available for NBM patients. The aim of this study was to create a computational network model simulating the different intracellular pathways involved in NBM, in order to predict how the tumor phenotype may be influenced to increase the sensitivity to anti-programmed cell death-ligand-1 (PD-L1)/programmed cell death-1 (PD-1) immunotherapy. The model runs on COPASI software. In order to determine the influence of intracellular signaling pathways on the expression of PD-L1 in NBM, we first developed an integrated network of protein kinase cascades. Michaelis–Menten kinetics were associated to each reaction in order to tailor the different enzymes kinetics, creating a system of ordinary differential equations (ODEs). The data of this study offers a first tool to be considered in the therapeutic management of the NBM patient undergoing immunotherapeutic treatment.

## 1. Introduction

Neuroblastoma (NBM) is one of the most frequent pediatric solid tumors. It accounts for 7% of malignancies diagnosed in children between ages from 0 to 14, and nearly 15% of pediatric cancer-related mortality [1]. Originating from neural crest cells, it shows heterogeneous clinical behaviors regarding maturation, regression, and aggressive growth [2]. The International Neuroblastoma Staging Series (INSS) stratifies NBM patients by risk level, tumor location, and dissemination and *MYCN* amplification [3].

A more recent classification from the International Neuroblastoma Risk Group (INRG) Task Force was made after collecting clinical data and bio-samples from more than 8800 cases in North America, Australia, Europe, and Japan [3].

The standard of care (SOC) for NBM consists of surgery, dose-intensive chemotherapy, radiation, and immunotherapeutic targeting of the disialoganglioside, GD2 [4,5]. The five-year survival rate for NBM is 79%. However, this survival rate depends on many factors, particularly the risk grouping of the tumor. In fact, while for children with low-risk NBM, the five-year survival rate is higher than 95%, it declines to 40%–50% for in high-risk NBM [6]. Hence, new therapeutic approaches are needed for this latter group of patients.

During the last few years a great body of preclinical and clinical data have proven that the immune system plays a key role in generation, maintenance, and growth of different tumors. In particular, it has been shown that immunosuppressive mechanisms, that operate in the tumor microenvironment, may favor the growth and metastatization of the tumor. The best described inhibitor immune checkpoints are represented by cytotoxic T lymphocyte–associated protein 4 (CTLA-4) and programmed cell death-1 (PD-1) [7]. It has been proven that monoclonal antibodies targeting these immune checkpoints, such as ipilimumab for CTLA-4 and nivolumab and pembrolizumab for PD-1, exert clear-cut beneficial effects on the clinical course of cancer, including metastatic melanoma, non-small cell lung cancer, renal cell carcinoma, and Hodgkin lymphoma [8,9,10].

Much attention has been lately posed to the immunological profile of NBM and on the possibility that development of an immunosuppressive microenvironment may be implicated in the development of more aggressive forms of NBM. Indeed, it has been demonstrated that the combination of programmed cell death-ligand-1 (PD-L1) and HLA (Human Leukocyte Antigen) class I represents a novel prognostic biomarker for NBM, as a high number of HLA class I-expressing tumor cells and low PD-L1 correlate with a better prognosis [11]. The use of computational modeling may offer a unique tool for the prediction of responses to immunotherapeutic treatments, as it is can be used to integrate robust data input, from genomic and transcriptomic studies, clinical data, and in vivo and in vitro experimental models. In NSCLC (Non-Small Cell Lung Cancer), immunotherapy has often proved to be a winning solution and computational models have been developed to give an estimation of responding patients. Brogden et al. developed a network model to evaluate the expression of PD-L1, used as an index of outcome following anti-PDL-1 therapy [12].

The aim of this work was to create a computational model for the prediction of the response of NBM patients to immunotherapeutic treatments. We first considered the pathways potentially affecting the expression of PDL-1 and we differentiated the cases in which there was or not the activating mutation of *ALKF1174L*. Afterwards, we analyzed the modulation of PDL-1 upon treatment with gefitinib (Epidermal Growth Factor Receptor -EGFR -inhibitor) or crizotinib (ALK- Anaplastic Lymphoma Kinase- inhibitors), alone or in combination.

## 2. Materials and Methods

### 2.1. Construction of a Signaling Network in NBM

In order to determine the influence of intracellular signaling pathways on the expression of PD-L1 in NBM, we first developed an integrated network of protein kinase cascades. The network was developed starting from models retrieved from the Kyoto Encyclopedia of Genes and Genomes (Kegg) PATHWAY database and it was manually curated based on the information retrieved from a survey of literature found on PubMed. The interaction between the PI3K (PhosphoInositide-3-Kinase)–AKT signaling pathway (Kegg reference: map04151), the MAPK (Mitogen-Activated Protein Kinase) signaling pathway (Kegg reference: map04010), the mTOR (mammalian Target Of Rapamycin) signaling pathway (Kegg reference: map04150), and the Ras signaling pathway (Kegg reference: map04014) were first considered. Based on the role of EGFR and NGFR (Nerve Growth Factor Receptor) in NBM, the neurotrophin signaling pathway (Kegg reference: map04150) and the EGFR tyrosine kinase inhibitor resistance (Kegg reference: map04722) were also included. Finally, the presence of the activating *ALK1174L* mutation was modeled in the network. The data were manually entered into the CellDesigner software (version 4.4; http://www.celldesigner.org/) for graphical representation.

### 2.2. Parameters Estimation and Kinetics Simulation

The signaling network was imported in COPASI (COmplex PAthway SImulator) (version 4.25; http://copasi.org/), a software for the simulation and analysis of biochemical networks and dynamics. The molecular reactions were modeled considering data obtained from the literature and can be categorized into post-translational modifications, activation/inhibition of proteins, protein degradation, and transcriptional regulation and translation. Transcription and translation phenomena, according to the literature, have been described by Henri–Michaelis–Menten equations, while degradation events have been modeled by considering the mass action law. The Henri–Michaelis–Menten equations describe the rate of enzymatic reactions, by relating the reaction rate to the concentration of a substrate S. The law of mass action assumes that the rate of a chemical reaction is directly proportional to the product of the activities or concentrations of the reactants. Data specific to each molecular event and PubMed identifiers of the annotated research articles are provided as Supplementary Material. The reactions that had not already been described in the literature were modeled according to modified Henri–Michaelis–Menten equations, in which the role of the activator/repressor was taken into account. Each reaction was described as an ordinary differential equation (ODE). Relative initial concentrations of each inactive species included in our model were gathered from RNASeq data from the TARGET 2018 Database of Pediatric NBM samples, obtained from cBioPortal, whereas the initial amount of the corresponding activated states was fixed to zero. For the determination of the kinetic rate constant of the Henri–Michaelis–Menten equations, we used the COPASI linear regression tool, that allowed to calculate the parameters best fitting with the experimental data. Simulations were calculated by running a deterministic algorithm (LSODA), considering a small and a large time scale. In the first case, we chose the following parameters: duration, 1 × 10^−5^ s; interval size, 2.328304971 × 10^−11^; intervals, 429,497; integrate reduced model, 0; relative tolerance, 1 × 10^−6^; absolute tolerance, 1 × 10^−12^; and maximum internal steps, 10. Whilst, in the second one, we used: duration, 2 × 10^5^ s; interval size, 4.656609941; intervals, 429,50; integrate reduced model, 0; relative tolerance, 1 × 10^−6^; absolute tolerance, 1 × 10^−12^; and maximum internal steps, 10. The entire list of the equations and reactions considered, the values of the calculated parameters and the relative references are provided as Supplementary Material.

### 2.3. Sensitivity Analysis

COPASI was also used for the calculation of sensitivities of the constructed model with respect to the imputed parameters. Sensitivity analysis allows to determine the parameters that are most involved in the regulation of one specific model species. We used this tool to evaluate how the levels of active PD-L1 were modulated with regards to the various kinetics parameters of the reactions considered. Given the complexity of our model, the network was simplified to 54 species and 43 reactions in order to perform the sensitivity analysis.

### 2.4. Validation Analysis

In order to validate the data obtained from the mathematical model, the GSE107354 microarray dataset was interrogated. For the generation of the dataset, the NB39nu neuroblastoma cell line, harboring ALK amplification, cultured in 2D and 3D conditions, was treated for 24 h with either DMSO or the ALK inhibitors, crizotinib and alectinib, at a final concentration of 1 μM, and RNA extracted and hybridized to the Agilent-039494 SurePrint G3 Human GE v2 × 8 × 60K microarrays. Array probes were logarithm transformed and normalized using the quantile method [13].

## 3. Results

### 3.1. Signaling Network in NBM and Sensitivity Analysis

**Figure 1:**
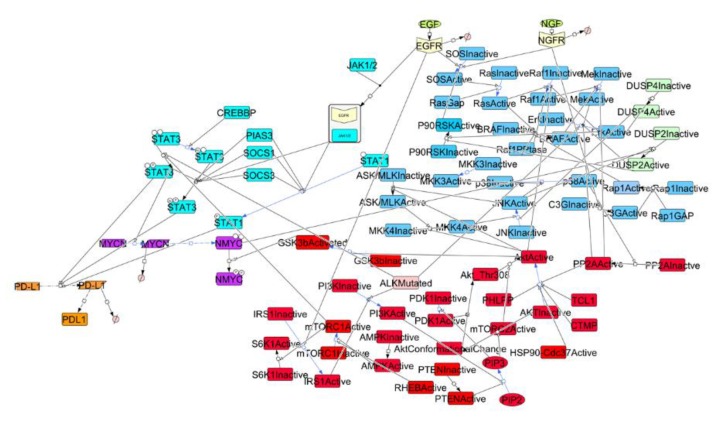
Graphic representation of the pathways considered in the model. Square shape: gene; diamond shape: mRNA; circle shape: simple molecule; square shape with smooth corners: protein; Ø: degraded; blue: MAPK (Mitogen-activated protein kinase) pathway, red: PI3K (phosphoinositide-3-kinase)/AKT/mTOR (mammalian Target Of Rapamycin) pathway; light blue: JAK (Janus Kinase)/STAT (Signal Transducer and Activator of Transcription) pathway; purple: MYCN transcription; orange: programmed cell death-ligand 1 (PD-L1) transcription.

We screened over 200 research articles and documented intracellular signaling reactions from 36 manuscripts. The annotated molecular events were graphically depicted using the CellDesigner tool (Version number, website), as presented in Figure 1. In the network, each node represented a molecule and each reaction was represented by an edge. The post-translational modifications annotated included phosphorylation, dephosphorylation, and acetylation. The upstream signal from EGF and NGF was included, as well as the signal from the active form of ALK, bearing the *ALK1174L* mutation. The network was constructed to have the PD-L1 expression as downstream node. Overall, our model comprised 93 species and 85 reactions. Each reaction was described by an ordinary differential equation (ODE), as shown in Supplementary Material. The model has been deposited in BioModels and assigned the identifier, MODEL1812070002. 

**Figure 2:**
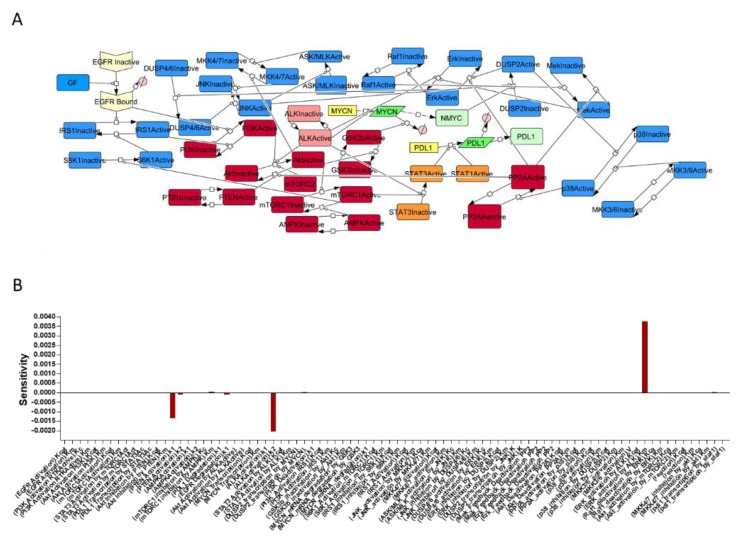
Simplified pathway used for the sensitivity analysis (**A**). The sensitivity analysis shows the influence of reaction parameters (Kcat, Km, k1 or k2) on PD-L1 transcription. Negative values indicate repression of transcription, while positive ones indicate an induction. The Km of ERK (Extracellular signal–Regulated kKinase) activation by ALK (Anaplastic Lymphoma Kinase) was the parameter most associated with PD-L1 expression. A strong negative value was elicited for k1 of PTEN (Phosphatase and Tensin homolog) activation and k2 of ALK activation, meaning that both the parameters were inversely proportional to PD-L1 expression (**B**).

We performed a sensitivity analysis in order to establish which parameters mostly affected the expression of PD-L1. Because of the complexity of the network generated, the steady state could not be reached, therefore a simplified version of the network was used (Figure 2A). The results from the analysis showed that the most important reactions for the regulation of PD-L1 expression were represented by ALK activation and AKT inhibition, and ERK (Extracellular signal–Regulated Kinase) activation (Figure 2B).

### 3.2. PDL-1 Expression is Controlled by ALK

**Figure 3:**
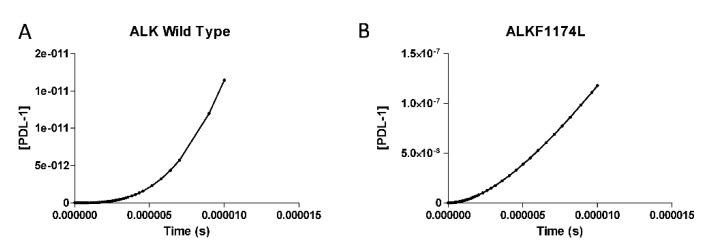
Expression of PD-L1 in a neuroblastoma cell line without ALK mutations (**A**). PD-L1 expression in a neuroblastoma cell line harboring *ALKF1174L* mutation (**B**). The simulation was conducted from 0 s to 1 × 10^−5^ s, using the deterministic (LSODA) method. All concentrations are in mmol/mL.

**Figure 4:**
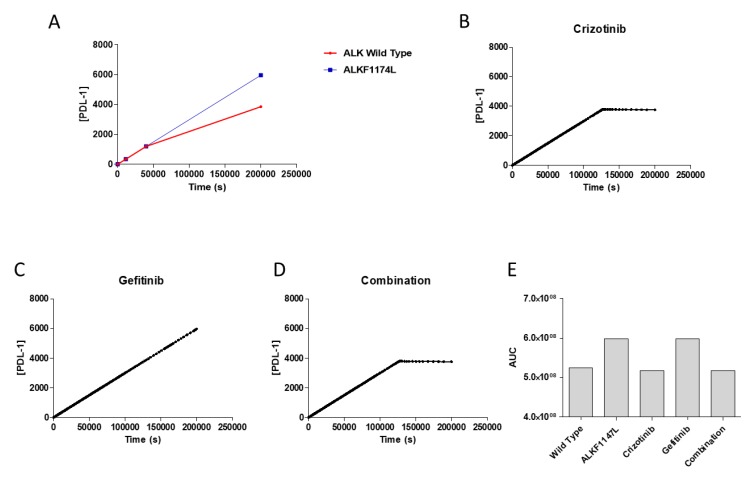
Expression of PD-L1 in a neuroblastoma cell line with and without ALK mutation (**A**). (**B**) Expression of PD-L1 after treatment with 1.4 × 10^−3^ mM crizotinib therapy (**C**), 3 × 10^−3^ mM gefitinib therapy (**D**) and a combination of the two inhibitors (**E**). The simulation was conducted from 0 to 2 × 10^5^ s., using the deterministic (LSODA) method. All concentrations are in mmol/mL. Comparison of the cumulative area under the curves (AUCs) of PD-L1 expression without therapy and with different therapy regimens (**F**).

We simulated our model under EGF and NGF stimulation conditions, in the presence or not of the *ALK1174L* mutation. As shown in Figure 3, in a time frame of 0–1 × 10^−5^ s, PD-L1 expression increased with a faster kinetics in the presence of the *ALK1174L* mutation. Indeed, the predicted concentration of PD-L1 mRNA at t = 1 × 10^−5^ s was 1.18 × 10^−7^ mmol/mL for the mutated tumor and 1.65 × 10^−11^ mmol/mL for the wild type tumor (Figure 3). In a longer time-frame (i.e., 0–2 × 10^5^ s), while in the non-mutated tumor, the concentration of PD-L1 reached its plateau concentration at t = 1.3 × 10^5^ s, in the *ALKF1174L* mutated tumor the concentration of PD-L1 continued to increase in a linear fashion (Figure 4A).

### 3.3. Effects of Pharmacological Treatment on PDL-1 Expression

Next, we simulated what happens upon specific target therapy, in particular with the use of crizotinib (ALK inhibitor) and gefitinib (EGFR inhibitor), as single and combination therapy. The model was run under EGF and NGF stimulation conditions in the *ALK1174L* mutated tumor. The ODEs implemented in the model were the following:

(1) $$\begin{array}{l} \frac{d\left(\left[ALK\_Mutated\right]\times V_{“\mathrm{Neuroblastoma}\;\mathrm{Cell}\;\mathrm{Cytoplasm}”}\right)}{dt}\\ =-V_{“\mathrm{Neuroblastoma}\;\mathrm{Cell}\;\mathrm{Cytoplasm}”}\\ \times HMM\_Modified\left(Kcat_{\left(crizotinib\right)},\;km_{\left(crizotinib\right)},\left[Crizotinib\right],\left[ALK\_Mutated\right]\right) \end{array}$$

(2) $$\begin{array}{l} \frac{d\left(\left[crizotinib\right]\times V_{“\mathrm{Neuroblastoma}\;\mathrm{Cell}\;\mathrm{Cytoplasm}”}\right)}{dt}\\ =-V_{“\mathrm{Neuroblastoma}\;\mathrm{Cell}\;\mathrm{Cytoplasm}”}\times K1_{\left(Crizotinib\;Degradation\right)}\\ \times \left[Crizotinib\right] \end{array}$$

(3) $$\begin{array}{l} \frac{d\left(\left(EGFR\_inhibited\right)\times V_{“\mathrm{Neuroblastoma}\;\mathrm{Cell}\;\mathrm{Cytoplasm}”}\right)}{dt}\\ =+V_{“\mathrm{Neuroblastoma}\;\mathrm{Cell}\;\mathrm{Cytoplasm}”}\times HMM\_Modified\\ \times \left({Kcat}_{\left({\mathrm{EGFR}}_{\mathrm{inhibitor}}\right)},{km}_{\left({\mathrm{EGFR}}_{\mathrm{inhibitor}}\right)},\left[Gefitinib\right],\left[\mathrm{EGFR}\_\mathrm{free}\right]\right) \end{array}$$

(4) $$\begin{array}{l} \frac{d\left(\left(Gefitinib\right)\times V_{“\mathrm{Neuroblastoma}\;\mathrm{Cell}\;\mathrm{Cytoplasm}”}\right)}{dt}\\ =-V_{“\mathrm{Neuroblastoma}\;\mathrm{Cell}\;\mathrm{Cytoplasm}”}\times K1_{\left(Gefitinib\;degradation\right)}\times \left[Gefitinib\right] \end{array}$$

In our model, the predicted peak concentration of PD-L1 upon crizotinib treatment was dramatically lower than that observed in the untreated cell (crizotinib: 3761.26mmol/mL; control: 5962.23 mmol/mL at t = 2 × 10^5^) (Figure 4). On the other hand, EGFR inhibition did not influence the levels of PD-L1, which reached the same concentration as in the control tumor (Figure 4). The combination therapy showed no differences in the expression levels of PD-L1 as compared to the crizotinib or gefitinib therapy alone (Figure 4).

### 3.4. Validation Analysis

**Figure 5:**
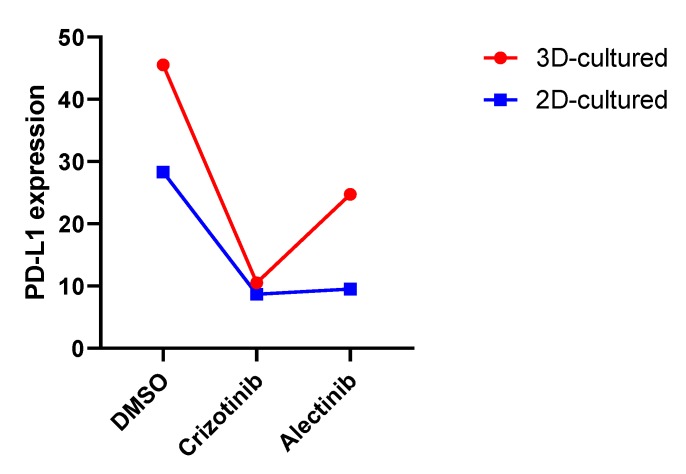
Effect of crizotinib and alectinib on PD-L1 expression in the neuroblastoma cell line, NB39nu, harboring ALK amplification, as determined in the GSE107354 dataset.

In order to validate the data obtained using COPASI analysis, the GSE107354 dataset was analyzed. As shown in Figure 5, treatment of the neuroblastoma cell line, NB39nu, with the ALK inhibitors, crizotinib and alectinib, was associated to a marked downregulation of PD-L1 expression. The effect was more evident upon treatment with crizotinib, which led to a 4.33-fold reduction in PD-L1 levels in 3D-cultured cells and a 3.26-fold reduction in 2D-cultured cells (Figure 5).

## 4. Discussion

NBM is a neoplastic transformation of undifferentiated sympatho–adrenal (SA) progenitor cells that results in tumor formation in the adrenal medulla and sympathetic ganglia. NBM cells metastasize to the bone marrow, bone, lymph nodes, liver, lung, central nervous system, and skin, resulting in long-term survival rates of less than 40%, even with intensive treatment. The first manifestation can be a loco-regional disease, with signs and symptoms of a space-occupying lesion, such as palpable abdominal or pelvic mass or spinal nerve compression, with related neurological symptoms. NBM displays the highest rate of spontaneous regression observed among human cancers, and one possible explanation for this phenomenon is the induction of patients’ immune responses toward tumor cells. Some authors detected a higher frequency of PD-L1+ tumor cells in high-risk NBM [14], whereas other authors reported no expression of PD-L1 in both primary and metastatic neuroblastoma lesions [15,16].

Anticancer immunity can be impaired by a variety of immunosuppressive mechanisms, including the expression of inhibitory checkpoints, such as PD-1, CTLA-4 and lymphocyte activation gene 3 (LAG3), which limit the effector function of T cells by interacting with their ligands, expressed on a wide range of tumor cells. Blockade of PD-1 and PD-L1 by monoclonal antibodies (mAbs) has provided encouraging results in a wide variety of cancers, such as melanoma [17], renal carcinoma [10], non-small cell lung cancer [18], and have been tested in a murine model of NBM [19].

However, it is not yet possible to determine how immunotherapy will affect a particular patient or whether the patient will respond or not.

Bioinformatics, machine learning-based modeling and drug repositioning-science can provide useful information on the molecular mechanisms driving NBM pathogenesis and help in the search of potential therapeutics [3,20]. The use of in silico analysis has been largely used by us to identify pathogenic pathway and novel therapeutic targets in a variety of clinical settings [21,22], including autoimmune diseases [23,24,25,26,27,28], cancer [22,29,30,31,32,33], fibrotic diseases [34], infectious diseases [35], and psychiatric and neurological disorders [36,37]. Mathematical modeling is helpful in predicting cell behaviors and uncovering how the dynamic interactions of signal transducers lead to context-dependent cellular responses [38]. To investigate the dynamic interactions between system components, four key properties are required: the system structure (individual components and interactions between them), the system dynamics (system behavior under different conditions and over time), the control methods (principles governing regulation of the system), and the design methods (principles governing system construction) [39]. This approach provides a deeper understanding of the tumor phenotype and may suggest how the system can be modulated in order to induce desired phenotypic changes.

Unlike adult cancers, which are characterized by many acquired somatic mutations, NBM exhibits surprisingly few recurrent mutations conserved across high-risk cases, as assessed in several large-scale sequencing studies [40]. Among the frequently mutated genes, activating mutations in the ALK kinase and loss-of-function mutations in the paired-like homeobox 2b (PHOX2B) transcription factor account for ~80% of hereditary neuroblastomas [1,40]. Recently, a cooperative oncogenic effect of MYCN and activated ALK in neuroblastomagenesis has been demonstrated [41,42].

We created a computational model in order to predict how the NBM tumor phenotype could be modulated to respond to anti-PD-1/PD-L1 therapy. In our study, we showed that ALK is a major player in the expression of PDL-1, and that treatment with crizotinib (ALK inhibitor) decreased the concentrations of PD-L1. On the other hand, in the *ALK1174L* mutated tumor, PD-L1 expression was not influenced by the anti-EGFR drug, gefitinib. These results suggest that patients bearing the *ALK1174L* mutation may probably benefit the most from a therapy with nivolumab. These considerations are very useful for determining which patients should be directed to a possible immunotherapy.

This study aims to provide a useful tool to be considered in the therapeutic management of NBM patients and, eventually, in the redaction of guidelines and in clinical practice, applying a targeted therapy, in the attempt to optimize the current therapeutic strategies and achieving the goal of personal medicine. Our model can also be useful for the development of predictive systems for other cancers, such as medulloblastoma, glioblastoma, melanoma, rhabdomyosarcoma and Wilms’ Tumor.

Future studies will expand the number of pathways considered in our network model and optimize the equations used to represent the interaction of genes involved. Afterward, it will be necessary to integrate the information gathered to make more precise predictions. An assessment of the potential interaction between cancer cells and immune system could also be modeled to design more effective therapeutic strategies.

## 5. Conclusions

Developing a mathematical model able to determine the factors influencing PD-L1 expression, and to a more general extent, predict the response to immunotherapy, will help in the future to design a potential clinical decision-support system, guiding the clinicians in the selection of therapies based on individual patient genomic profiles. Although the model here described requires extensive implementation and should be validated in the clinical setting, its utility in the clinical practice is possible, as a tool to achieve a targeted-tailored and personalized medicine.

## Supplementary Materials

The following are available online at https://www.mdpi.com/2076-3425/9/9/221/s1.

## References

[1] Maris J.M., Hogarty M.D., Bagatell R., Cohn S.L. (2007). Neuroblastoma. Lancet.

[2] Bolande R.P. (1974). The neurocristopathiesA unifying concept of disease arising in neural crest maldevelopment. Hum. Pathol..

[3] Salazar B.M., Balczewski E.A., Ung C.Y., Zhu S. (2017). Neuroblastoma, a paradigm for big data science in pediatric oncology. Int. J. Mol. Sci..

[4] Maris J.M. (2012). Recent advances in neuroblastoma. N. Engl. J. Med..

[5] Yu A.L., Gilman A.L., Ozkaynak M.F., London W.B., Kreissman S.G., Chen H.X., Smith M., Anderson B., Villablanca J.G., Matthay K.K. (2011). Anti-GD2 Antibody with GM-CSF, Interleukin-2, and Isotretinoin for Neuroblastoma. N. Engl. J. Med..

[6] Richards R.M., Sotillo E., Majzner R.G. (2018). CAR T Cell Therapy for Neuroblastoma. Front. Immunol..

[7] D’arrigo P., Tufano M., Rea A., Vigorito V., Novizio N., Russo S., Romano M.F., Romano S. (2018). Manipulation of the immune system for cancer defeat: A focus on the T cell inhibitory checkpoint molecules. Curr. Med. Chem..

[8] Larkin J., Chiarion-Sileni V., González R., Grob J., Cowey C., Lao C. (2015). Combined Nivolumab and Ipilimumab or Monotherapy in Previously Untreated Melanoma. N. Engl. J. Med..

[9] Ritprajak P., Azuma M. (2014). Intrinsic and extrinsic control of expression of the immunoregulatory molecule PD-L1 in epithelial cells and squamous cell carcinoma Intrinsic and extrinsic control of expression of the immunoregulatory molecule PD-L1 in epithelial cells and squamous cell. ORAL Oncol..

[10] Massari F., Santoni M., Ciccarese C., Santini D., Alfieri S., Martignoni G., Brunelli M., Piva F., Berardi R., Montironi R. (2015). PD-1 blockade therapy in renal cell carcinoma: Current studies and future promises. Cancer Treat. Rev..

[11] Melaiu O., Mina M., Chierici M., Boldrini R., Jurman G., Romania P., D’Alicandro V., Benedetti M.C., Castellano A., Liu T. (2017). PD-L1 Is a Therapeutic Target of the Bromodomain Inhibitor JQ1 and, Combined with HLA Class I, a Promising Prognostic Biomarker in Neuroblastoma. Clin. Cancer Res..

[12] Brogden K.A., Parashar D., Hallier A.R., Braun T., Qian F., Rizvi N.A., Bossler A.D., Milhem M.M., Chan T.A., Abbasi T. (2018). Genomics of NSCLC patients both affirm PD-L1 expression and predict their clinical responses to anti-PD-1 immunotherapy. BMC Cancer.

[13] Miyazaki M., Otomo R., Matsushima-Hibiya Y., Suzuki H., Nakajima A., Abe N., Tomiyama A., Ichimura K., Matsuda K., Watanabe T. (2018). The p53 activator overcomes resistance to ALK inhibitors by regulating p53-target selectivity in ALK-driven neuroblastomas. Cell Death Discov..

[14] Chowdhury F., Dunn S., Mitchell S., Mellows T., Ashton-Key M., Gray J.C. (2015). PD-L1 and CD8+PD1+ lymphocytes exist as targets in the pediatric tumor microenvironment for immunomodulatory therapy. Oncoimmunology.

[15] Aoki T., Hino M., Koh K., Kyushiki M., Kishimoto H., Arakawa Y., Hanada R., Kawashima H., Kurihara J., Shimojo N. (2016). Low Frequency of Programmed Death Ligand 1 Expression in Pediatric Cancers. Pediatr. Blood Cancer..

[16] Dondero A., Pastorino F., Chiesa M.D., Corrias M.V., Morandi F., Pistoia V., Olive D., Bellora F., Locatelli F., Castellano A. (2016). PD-L1 expression in metastatic neuroblastoma as an additional mechanism for limiting immune surveillance. Oncoimmunology.

[17] Ott P.A., Hodi F.S., Robert C. (2013). CTLA-4 and PD-1/PD-L1 Blockade: New Immunotherapeutic Modalities with Durable Clinical Benefit in Melanoma Patients. Clin. Cancer Res..

[18] Akbay E.A., Koyama S., Carretero J., Altabef A., Tchaicha J.H., Christensen C.L., Mikse O.R., Cherniack A.D., Beauchamp E.M., Pugh T.J. (2013). Activation of the PD-1 pathway contributes to immune escape in EGFR-driven lung tumors. Cancer Discov..

[19] Srinivasan P., Wu X., Basu M., Rossi C., Sandler A.D. (2018). PD-L1 checkpoint inhibition and anti-CTLA-4 whole tumor cell vaccination counter adaptive immune resistance: A mouse neuroblastoma model that mimics human disease. PLoS Med..

[20] Azuaje F. (2017). Computational models for predicting drug responses in cancer research. Brief. Bioinform..

[21] Mammana S., Fagone P., Cavalli E., Basile M.S., Petralia M.C., Nicoletti F., Bramanti P., Mazzon E. (2018). The Role of Macrophages in Neuroinflammatory and Neurodegenerative Pathways of Alzheimer’s Disease, Amyotrophic Lateral Sclerosis, and Multiple Sclerosis: Pathogenetic Cellular Effectors and Potential Therapeutic Targets. Int. J. Mol. Sci..

[22] Mangano K., Mazzon E., Basile M.S., Di Marco R., Bramanti P., Mammana S., Petralia M.C., Fagone P., Nicoletti F. (2018). Pathogenic role for macrophage migration inhibitory factor in glioblastoma and its targeting with specific inhibitors as novel tailored therapeutic approach. Oncotarget.

[23] Fagone P., Mazzon E., Cavalli E., Bramanti A., Petralia M.C., Mangano K., Al-Abed Y., Bramati P., Nicoletti F. (2018). Contribution of the macrophage migration inhibitory factor superfamily of cytokines in the pathogenesis of preclinical and human multiple sclerosis: In silico and in vivo evidences. J. Neuroimmunol..

[24] Mangano K., Cavalli E., Mammana S., Basile M.S., Caltabiano R., Pesce A., Puleo S., Atanasov A.G., Magro G., Nicoletti F. (2018). Involvement of the Nrf2/HO-1/CO axis and therapeutic intervention with the CO-releasing molecule CORM-A1, in a murine model of autoimmune hepatitis. J. Cell. Physiol..

[25] Mammana S., Bramanti P., Mazzon E., Cavalli E., Basile M.S., Fagone P., Petralia M.C., McCubrey J.A., Nicoletti F., Mangano K. (2018). Preclinical evaluation of the PI3K/Akt/mTOR pathway in animal models of multiple sclerosis. Oncotarget.

[26] Fagone P., Muthumani K., Mangano K., Magro G.G., Meroni P.L., Kim J.J., Sardesai N.Y., Weiner D.B., Nicoletti F. (2014). VGX-1027 modulates genes involved in lipopolysaccharide-induced Toll-like receptor 4 activation and in a murine model of systemic lupus erythematosus. Immunology.

[27] Fagone P., Mazzon E., Mammana S., Di Marco R., Spinasanta F., Basile M.S., Petralia M.C., Bramanti P., Nicoletti F., Mangano K. (2019). Identification of CD4+ T cell biomarkers for predicting the response of patients with relapsing-remitting multiple sclerosis to natalizumab treatment. Mol. Med. Rep..

[28] Nicoletti F., Mazzon E., Fagone P., Mangano K., Mammana S., Cavalli E., Basile M.S., Bramanti P., Scalabrino G., Lange A. (2019). Prevention of clinical and histological signs of MOG-induced experimental allergic encephalomyelitis by prolonged treatment with recombinant human EGF. J. Neuroimmunol..

[29] Presti M., Mazzon E., Basile M.S., Petralia M.C., Bramanti A., Colletti G., Bramanti P., Nicoletti F., Fagone P. (2018). Overexpression of macrophage migration inhibitory factor and functionally-related genes, D-DT, CD74, CD44, CXCR2 and CXCR4, in glioblastoma. Oncol. Lett..

[30] Fagone P., Caltabiano R., Russo A., Lupo G., Anfuso C.D., Basile M.S., Longo A., Nicoletti F., De Pasquale R., Libra M. (2017). Identification of novel chemotherapeutic strategies for metastatic uveal melanoma. Sci. Rep..

[31] Basile M.S., Mazzon E., Russo A., Mammana S., Longo A., Bonfiglio V., Fallico M., Caltabiano R., Fagone P., Nicoletti F. (2019). Differential modulation and prognostic values of immune-escape genes in uveal melanoma. PLoS ONE.

[32] Petralia M.C., Mazzon E., Fagone P., Russo A., Longo A., Avitabile T., Nicoletti F., Reibaldi M., Basile M.S. (2019). Characterization of the Pathophysiological Role of CD47 in Uveal Melanoma. Molecules.

[33] Candido S., Lupo G., Pennisi M., Basile M.S., Anfuso C.D., Petralia M.C., Gattuso G., Vivarelli S., Spandidos D.A., Libra M. (2019). The analysis of miRNA expression profiling datasets reveals inverse microRNA patterns in glioblastoma and Alzheimer’s disease. Oncol. Rep..

[34] Fagone P., Mangano K., Mammana S., Pesce A., Pesce A., Caltabiano R., Giorlandino A., Portale T.R., Cavalli E., Lombardo G.A. (2015). Identification of novel targets for the diagnosis and treatment of liver fibrosis. Int. J. Mol. Med..

[35] Fagone P., Nunnari G., Lazzara F., Longo A., Cambria D., Distefano G., Palumbo M., Nicoletti F., Malaguarnera L., Di Rosa M. (2016). Induction of OAS gene family in HIV monocyte infected patients with high and low viral load. Antivir. Res..

[36] Petralia M.C., Mazzon E., Fagone P., Falzone L., Bramanti P., Nicoletti F., Basile M.S. (2019). Retrospective follow-up analysis of the transcriptomic patterns of cytokines, cytokine receptors and chemokines at preconception and during pregnancy, in women with post-partum depression. Exp. Ther. Med..

[37] Lombardo S.D., Mazzon E., Basile M.S., Cavalli E., Bramanti P., Nania R., Fagone P., Nicoletti F., Petralia M.C. (2019). Upregulation of IL-1 Receptor Antagonist in a Mouse Model of Migraine. Brain Sci..

[38] Arkin A.P., Schaffer D.V. (2011). Network News: Innovations in 21st Century Systems Biology. Cell.

[39] Kitano H. (2002). Systems Biology: A Brief Overview. Science.

[40] Speleman F., De Preter K., Vandesompele J. (2011). Neuroblastoma genetics and phenotype: A tale of heterogeneity. Semin. Cancer Boil..

[41] Diskin S.J., Capasso M., Schnepp R.W., A Cole K., Attiyeh E.F., Hou C., Diamond M., Carpenter E.L., Winter C., Lee H. (2012). Common variation at 6q16 within HACE1 and LIN28B influences susceptibility to neuroblastoma. Nat. Genet..

[42] Sausen M., Leary R.J., Jones S., Wu J., Reynolds C.P., Liu X., Blackford A., Parmigiani G., Diaz L.A., Papadopoulos N. (2013). Integrated genomic analyses identify ARID1A and ARID1B alterations in the childhood cancer neuroblastoma. Nat. Genet..

