# Supplementary material for: Prediction of PD-L1 Expression in Neuroblastoma via Computational Modeling
Source: Brain Sci. 2019 Aug 31;9(9):221. doi: 10.3390/brainsci9090221 (PMC6770763; doi:10.3390/brainsci9090221)
Supplement: Supplementary file 1 [file brainsci-09-00221-s001.pdf]

(EGFR Activation).Kcat (EGFR Activation).Km (PI3K Activation RTKActive).Kcat (PI3K Activation RTKActive).Km (Akt Activation PI3K).Kcat (Akt Activation PI3K).Km (mTORC1Activation).Kcat (mTORC1Activation).Km (STAT3 Activation by mTORC1Active).Kcat (STAT3 Activation by mTORC1Active).Km (PDL1 Transcription by STAT3Active).Kcat (PDL1 Transcription by STAT3Active).Km (Akt Inhibition by PTEN).Kcat (Akt Inhibition by PTEN).Km (PTEN Activation).k1 (PTEN Activation).k2 (AMPKActivation).k1 (AMPKActivation).k2 (mTORC1 inhibition by AMPK).Kcat (mTORC1 inhibition by AMPK).Km (PDL1 Degradation).k1 (EGFR Degradation).k1 (Akt Activation by ALKActive).Kcat (Akt Activation by ALKActive).Km (MYCN Transcription).Kcat (MYCN Transcription).Km (ALKActivation).k1 (ALKActivation).k2 (STAT3 Activation by ALK).Kcat (STAT3 Activation by ALK).Km (DUSP2_transcription_by_MYCN).Kcat (DUSP2_transcription_by_MYCN).Km (PI3K_Deactivation).k1 (PI3K_Activation_IRS1).Kcat (PI3K_Activation_IRS1).Km (GSK3b_inactivation_by_Akt).Kcat (GSK3b_inactivation_by_Akt).Km (MYCN_mRNA_degradation_by_GSK3b).Kcat (MYCN_mRNA_degradation_by_GSK3b).Km (MYCN_mRNA_degradation).k1 (S6K1_Activation_mTORC1).Kcat (S6K1_Activation_mTORC1).Km (IRS1_Inhibition_by_S6K1).Kcat (IRS1_Inhibition_by_S6K1).Km (IRS1_Activation).Kcat (IRS1_Activation).Km (JNK_activation_by_MKK4/7).Kcat (JNK_activation_by_MKK4/7).Km (MKK4/7_activation).Kcat (MKK4/7_activation).Km (ASK/MLK_activation_by_JNK).Kcat (ASK/MLK_activation_by_JNK).Km (JNK_inhibition_by_DUSP4/6).Kcat (JNK_inhibition_by_DUSP4/6).Km (DUSP4/6_activation_by_ERK).Kcat (DUSP4/6_activation_by_ERK).Km (ERK_inhibition_by_DUSP2).Kcat (ERK_inhibition_by_DUSP2).Km (DUSP2_transcription_by_JNK).Kcat (DUSP2_transcription_by_JNK).Km (Mek_Feedback_Deactivation_PP2A).Kcat (Mek_Feedback_Deactivation_PP2A).Km (Erk_Feedback_Deactivation_PP2A).Kcat (Erk_Feedback_Deactivation_PP2A).Km (Akt_Deactivation_PP2A).Kcat (Akt_Deactivation_PP2A).Km (PP2A_activation_by_p38).Kcat (PP2A_activation_by_p38).Km (p38_activation).Kcat (p38_activation).Km (p38_inhibition_by_DUSP2).Kcat (p38_inhibition_by_DUSP2).Km(MKK3/6_activation).Km (MKK3/6_activation).V (ERK_activation by ALK).Kcat(ERK_activation by ALK).Km (Raf1_deactivation_by_ERK).Kcat (Raf1_deactivation_by_ERK).Km (Akt_activation_by_mTORC2).Kcat (Akt_activation_by_mTORC2).Km (reaction).Kcat (reaction).Km (MKK4/7_inhibition_by_akt).Kcat (MKK4/7_inhibition_by_akt).Km (pdl1_transcrtiption_by_stat1).Km (pdl1_transcrtiption_by_stat1).V

[PDL1Active] -1,57E-09 2,08E-07 -3,15E-12 -9,54E-08 -8,55E-13 -5,72E-10 0 0 0 -4,54E-11 4,84E-08 -2,21E-06 -3,14E-08 -1,28E-13 -1,34E-03 -7,56E-05 -2,31E-09 7,30E-08 4,55E-07 5,73E-05 nan -8,88E-05 -2,96E-07 -6,50E-12 -1,02E-12 0 -3,20E-11 -2,03E-03 1,63E-07 -1,43E-08 0 3,11E-05 -4,44E-06 0 0 0 0 0 0 -2,51E-06 0 0 0 0 0 0 0 0 0 0 0 0 0 0 -1,25E-12 -5,66E-07 0 0 0 0 0 0 0 0 -1,76E-07 4,18E-11 0 3,67E-14 -5,07E-11

1,54E-06 0 0 0 6,17E-10 3,85E-11 3,75E-03 0
0 0 -1,49E-08 0 0 0 0 2,43E-05 -5,76E-10

$$\frac{d\left([\text{ALK\_Mutated}] \cdot V_{\text{"Neuroblastoma Cell Cytoplasm"}}\right)}{dt} = -V_{\text{"Neuroblastoma Cell Cytoplasm"}} \cdot \text{HMM\_Modified}\left(\text{Kcat}_{(\text{Crizotinib})}, \text{km}_{(\text{Crizotinib})}, [\text{Crizotinib}], [\text{ALK\_Mutated}]\right)$$

$$\frac{d\left([\text{Crizotinib}] \cdot V_{\text{"Neuroblastoma Cell Cytoplasm"}}\right)}{dt} = -V_{\text{"Neuroblastoma Cell Cytoplasm"}} \cdot \text{k1}_{(\text{"Crizotinib Degradation"})} \cdot [\text{Crizotinib}]$$

$$\frac{d\left([\text{EGFR\_inhibited}] \cdot V_{\text{"Neuroblastoma Cell Cytoplasm"}}\right)}{dt} = +V_{\text{"Neuroblastoma Cell Cytoplasm"}} \cdot \text{HMM\_Modified}\left(\text{Kcat}_{(\text{EGFR\_inhibitor})}, \text{km}_{(\text{EGFR\_inhibitor})}, [\text{Gefitinib}], [\text{EGFR\_free}]\right)$$

$$\frac{d\left([\text{Gefitinib}] \cdot V_{\text{"Neuroblastoma Cell Cytoplasm"}}\right)}{dt} = -V_{\text{"Neuroblastoma Cell Cytoplasm"}} \cdot \text{k1}_{(\text{"Gefitinib degradation"})} \cdot [\text{Gefitinib}]$$

$$\frac{d\left([\text{EGFR\_free}]\cdot V_{\text{"Neuroblastoma Cell Cytoplasm"}}\right)}{dt} = -\left(k1_{(\text{"Biding EGF with EGFR"})}\cdot[\text{EGF}]\cdot[\text{EGFR\_free}] - k2_{(\text{"Biding EGF with EGFR"})}\cdot[\text{EGFR\_bound}]\right) - V_{\text{"Neuroblastoma Cell Cytoplasm"}}\cdot k1_{(\text{"EGFR\_free degradation"})}\cdot[\text{EGFR\_free}]$$

$$\frac{d\left([\text{EGFR\_bound}]\cdot V_{\text{"Neuroblastoma Cell Cytoplasm"}}\right)}{dt} = +\left(k1_{(\text{"Biding EGF with EGFR"})}\cdot[\text{EGF}]\cdot[\text{EGFR\_free}] - k2_{(\text{"Biding EGF with EGFR"})}\cdot[\text{EGFR\_bound}]\right) - V_{\text{"Neuroblastoma Cell Cytoplasm"}}\cdot k1_{(\text{EGFR\_bound\_degradation})}\cdot[\text{EGFR\_bound}]$$

$$\begin{aligned}\frac{d\left([\text{SOS\_active}]\cdot V_{\text{"Neuroblastoma Cell Cytoplasm"}}\right)}{dt} = &+V_{\text{"Neuroblastoma Cell Cytoplasm"}}\cdot\text{"SOS activation by EGFR"}(\text{KcatEGF}, [\text{EGFR\_bound}], [\text{SOS\_inactive}], \text{KmEGF})\\ &+V_{\text{"Neuroblastoma Cell Cytoplasm"}}\cdot\text{"SOS activation by NGF"}(\text{KcatNGF}, [\text{NGFR\_bound}], [\text{SOS\_inactive}], \text{KmNGF})\\ &-V_{\text{"Neuroblastoma Cell Cytoplasm"}}\cdot\text{"SOS deactivation by P90Rsk"}(\text{KcatSOS}, [\text{P90Rsk\_active}], [\text{SOS\_active}], \text{KmSOS})\\ &-V_{\text{"Neuroblastoma Cell Cytoplasm"}}\cdot\text{"SOS deactivation by ERK"}(\text{KcatERK}, [\text{ERK\_active}], [\text{SOS\_active}], \text{KmERK})\end{aligned}$$

$$\begin{aligned}\frac{d\left([\text{SOS\_inactive}]\cdot V_{\text{"Neuroblastoma Cell Cytoplasm"}}\right)}{dt} = &-V_{\text{"Neuroblastoma Cell Cytoplasm"}}\cdot\text{"SOS activation by EGFR"}(\text{KcatEGF}, [\text{EGFR\_bound}], [\text{SOS\_inactive}], \text{KmEGF})\\ &-V_{\text{"Neuroblastoma Cell Cytoplasm"}}\cdot\text{"SOS activation by NGF"}(\text{KcatNGF}, [\text{NGFR\_bound}], [\text{SOS\_inactive}], \text{KmNGF})\\ &+V_{\text{"Neuroblastoma Cell Cytoplasm"}}\cdot\text{"SOS deactivation by P90Rsk"}(\text{KcatSOS}, [\text{P90Rsk\_active}], [\text{SOS\_active}], \text{KmSOS})\\ &+V_{\text{"Neuroblastoma Cell Cytoplasm"}}\cdot\text{"SOS deactivation by ERK"}(\text{KcatERK}, [\text{ERK\_active}], [\text{SOS\_active}], \text{KmERK})\end{aligned}$$

$$\frac{d\left([\text{NGFR\_bound}]\cdot V_{\text{"Neuroblastoma Cell Cytoplasm"}}\right)}{dt} = +\left(k1_{(\text{"Biding NGF"})}\cdot[\text{NGF}]\cdot[\text{NGFR}] - k2_{(\text{"Biding NGF"})}\cdot[\text{NGFR\_bound}]\right) - V_{\text{"Neuroblastoma Cell Cytoplasm"}}\cdot k1_{(\text{"bNGFR degradation"})}\cdot[\text{NGFR\_bound}]$$

$$\frac{d\left([\text{NGFR}]\cdot V_{\text{"Neuroblastoma Cell Cytoplasm"}}\right)}{dt} = -\left(k1_{(\text{"Biding NGF"})}\cdot[\text{NGF}]\cdot[\text{NGFR}] - k2_{(\text{"Biding NGF"})}\cdot[\text{NGFR\_bound}]\right) - V_{\text{"Neuroblastoma Cell Cytoplasm"}}\cdot k1_{(\text{"NGFR degradation"})}\cdot[\text{NGFR}]$$

$$\frac{d\left([\text{P90Rsk\_active}]\cdot V_{\text{"Neuroblastoma Cell Cytoplasm"}}\right)}{dt} = -V_{\text{"Neuroblastoma Cell Cytoplasm"}}\cdot k1_{(\text{P90Rsk\_deactivation})}\cdot[\text{P90Rsk\_active}] + V_{\text{"Neuroblastoma Cell Cytoplasm"}}\cdot\text{"P90Rsk Activation"}(\text{KcatP90Rsk}, [\text{ERK\_active}], [\text{P90Rsk\_inactive}], \text{KmP90Rsk})$$

$$\frac{d\left([\text{P90Rsk\_inactive}]\cdot V_{\text{"Neuroblastoma Cell Cytoplasm"}}\right)}{dt} = +V_{\text{"Neuroblastoma Cell Cytoplasm"}}\cdot k1_{(\text{P90Rsk\_deactivation})}\cdot[\text{P90Rsk\_active}] - V_{\text{"Neuroblastoma Cell Cytoplasm"}}\cdot\text{"P90Rsk Activation"}(\text{KcatP90Rsk}, [\text{ERK\_active}], [\text{P90Rsk\_inactive}], \text{KmP90Rsk})$$

$$\frac{d\left([\text{RAS\_active}]\cdot V_{\text{"Neuroblastoma Cell Cytoplasm"}}\right)}{dt} = +V_{\text{"Neuroblastoma Cell Cytoplasm"}}\cdot\text{"RAS Activation"}(\text{KcatRAS}, [\text{SOS\_active}], [\text{RAS\_inactive}], \text{KmRAS}) - V_{\text{"Neuroblastoma Cell Cytoplasm"}}\cdot\text{"HMM modified"}(\text{KcatRasGap}, [\text{RasGap\_active}], [\text{RAS\_active}], \text{KmRasGap})$$

$$\frac{d\left([\text{RAS\_inactive}]\cdot V_{\text{"Neuroblastoma Cell Cytoplasm"}}\right)}{dt} = -V_{\text{"Neuroblastoma Cell Cytoplasm"}}\cdot\text{"RAS Activation"}(\text{KcatRAS}, [\text{SOS\_active}], [\text{RAS\_inactive}], \text{KmRAS}) + V_{\text{"Neuroblastoma Cell Cytoplasm"}}\cdot\text{"HMM modified"}(\text{KcatRasGap}, [\text{RasGap\_active}], [\text{RAS\_active}], \text{KmRasGap})$$

$$\frac{d\left([\text{PTEN\_active}]\cdot V_{\text{"Neuroblastoma Cell Cytoplasm"}}\right)}{dt} = +V_{\text{"Neuroblastoma Cell Cytoplasm"}}\cdot\left(k1_{(\text{PTEN\_activation})}\cdot[\text{PTEN\_inactive}] - k2_{(\text{PTEN\_activation})}\cdot[\text{PTEN\_active}]\right)$$

$$\frac{d\left([\text{S6K1\_inactive}]\cdot V_{\text{"Neuroblastoma Cell Cytoplasm"}}\right)}{dt} = -V_{\text{"Neuroblastoma Cell Cytoplasm"}}\cdot\text{HMM\_Modified}\left(\text{Kcat}_{(\text{S6K1\_Activation\_mTORC1})}, \text{km}_{(\text{S6K1\_Activation\_mTORC1})}, [\text{mTORC1\_active}], [\text{S6K1\_inactive}]\right)$$

$$\frac{d\left([\text{mTORC1\_inactive}]\cdot V_{\text{"Neuroblastoma Cell Cytoplasm"}}\right)}{dt} = -V_{\text{"Neuroblastoma Cell Cytoplasm"}}\cdot\text{HMM\_Modified}\left(\text{Kcat}_{(\text{mTORC1\_Activation\_RHEB})}, \text{km}_{(\text{mTORC1\_Activation\_RHEB})}, [\text{RHEB}], [\text{mTORC1\_inactive}]\right) + V_{\text{"Neuroblastoma Cell Cytoplasm"}}\cdot\text{HMM\_Modified}\left(\text{Kcat}_{(\text{mTORC1\_inhibition\_by\_AMPK})}, \text{km}_{(\text{mTORC1\_inhibition\_by\_AMPK})}, [\text{AMPK\_active}], [\text{mTORC1\_active}]\right)$$

$$\frac{d\left([\text{PDK1\_inactive}]\cdot V_{\text{"Neuroblastoma Cell Cytoplasm"}}\right)}{dt} = -V_{\text{"Neuroblastoma Cell Cytoplasm"}}\cdot\text{HMM\_Modified}\left(\text{Kcat}_{(\text{PDK1\_Activation})}, \text{km}_{(\text{PDK1\_Activation})}, [\text{PIP3}], [\text{PDK1\_inactive}]\right) + V_{\text{"Neuroblastoma Cell Cytoplasm"}}\cdot k1_{(\text{PDK1\_Deactivation})}\cdot[\text{PDK1\_active}]$$

$$\frac{d\left([\text{IRS1\_inactive}]\cdot V_{\text{"Neuroblastoma Cell Cytoplasm"}}\right)}{dt} = +V_{\text{"Neuroblastoma Cell Cytoplasm"}}\cdot\text{HMM\_Modified}\left(\text{Kcat}_{(\text{IRS1\_Feedback\_Deactivation\_S6K1})}, \text{km}_{(\text{IRS1\_Feedback\_Deactivation\_S6K1})}, [\text{S6K1\_active}], [\text{IRS1\_active}]\right) - V_{\text{"Neuroblastoma Cell Cytoplasm"}}\cdot\text{HMM\_Modified}\left(\text{Kcat}_{(\text{IRS1\_Activation})}, \text{km}_{(\text{IRS1\_Activation})}, [\text{EGFR\_bound}], [\text{IRS1\_inactive}]\right)$$

$$\frac{d\left([\text{PDK1\_active}]\cdot V_{\text{"Neuroblastoma Cell Cytoplasm"}}\right)}{dt} = +V_{\text{"Neuroblastoma Cell Cytoplasm"}}\cdot\text{HMM\_Modified}\left(\text{Kcat}_{(\text{PDK1\_Activation})}, \text{km}_{(\text{PDK1\_Activation})}, [\text{PIP3}], [\text{PDK1\_inactive}]\right) - V_{\text{"Neuroblastoma Cell Cytoplasm"}}\cdot k1_{(\text{PDK1\_Deactivation})}\cdot[\text{PDK1\_active}]$$

$$\frac{d\left([\text{mTORC1\_active}]\cdot V_{\text{"Neuroblastoma Cell Cytoplasm"}}\right)}{dt} = +V_{\text{"Neuroblastoma Cell Cytoplasm"}}\cdot\text{HMM\_Modified}\left(\text{Kcat}_{(\text{mTORC1\_Activation\_RHEB})}, \text{km}_{(\text{mTORC1\_Activation\_RHEB})}, [\text{RHEB}], [\text{mTORC1\_inactive}]\right) - V_{\text{"Neuroblastoma Cell Cytoplasm"}}\cdot\text{HMM\_Modified}\left(\text{Kcat}_{(\text{mTORC1\_inhibition\_by\_AMPK})}, \text{km}_{(\text{mTORC1\_inhibition\_by\_AMPK})}, [\text{AMPK\_active}], [\text{mTORC1\_active}]\right)$$

$$\frac{d\left([\text{S6K1\_active}]\cdot V_{\text{"Neuroblastoma Cell Cytoplasm"}}\right)}{dt} = +V_{\text{"Neuroblastoma Cell Cytoplasm"}}\cdot\text{HMM\_Modified}\left(\text{Kcat}_{(\text{S6K1\_Activation\_mTORC1})}, \text{km}_{(\text{S6K1\_Activation\_mTORC1})}, [\text{mTORC1\_active}], [\text{S6K1\_inactive}]\right)$$

$$\frac{d([IRS1\_active] \cdot V_{\text{"Neuroblastoma Cell Cytoplasm"}})}{dt} = -V_{\text{"Neuroblastoma Cell Cytoplasm"}} \cdot \text{HMM\_Modified}(Kcat_{(IRS1\_Feedback\_Deactivation\_S6K1)}, km_{(IRS1\_Feedback\_Deactivation\_S6K1)}, [S6K1\_active], [IRS1\_active]) + V_{\text{"Neuroblastoma Cell Cytoplasm"}} \cdot \text{HMM\_Modified}(Kcat_{(IRS1\_Activation)}, km_{(IRS1\_Activation)}, [EGFR\_bound], [IRS1\_inactive])$$

$$\frac{d([Akt\_inactive] \cdot V_{\text{"Neuroblastoma Cell Cytoplasm"}})}{dt} = -V_{\text{"Neuroblastoma Cell Cytoplasm"}} \cdot \text{HMM\_Modified}(Kcat_{(Akt\_Activation\_PIP3)}, km_{(Akt\_Activation\_PIP3)}, [PIP3], [Akt\_inactive]) + V_{\text{"Neuroblastoma Cell Cytoplasm"}} \cdot \text{HMM\_Modified}(Kcat_{(Akt\_Deactivation\_PP2A)}, km_{(Akt\_Deactivation\_PP2A)}, [PP2A\_active], [Akt\_active]) + V_{\text{"Neuroblastoma Cell Cytoplasm"}} \cdot \text{HMM\_Modified}(Kcat_{(Akt\_Deactivation\_PHLPP)}, km_{(Akt\_Deactivation\_PHLPP)}, [PHLPP], [Akt\_active]) + V_{\text{"Neuroblastoma Cell Cytoplasm"}} \cdot \text{HMM\_Modified}(Kcat_{(Akt\_Deactivation\_CTMP)}, km_{(Akt\_Deactivation\_CTMP)}, [CTMP], [Akt\_active]) - V_{\text{"Neuroblastoma Cell Cytoplasm"}} \cdot \text{HMM\_Modified}(Kcat_{(\text{"Akt\_Activation\_HSP90-Cdc37"})}, km_{(\text{"Akt\_Activation\_HSP90-Cdc37"})}, [\text{"HSP90-Cdc37Active"}], [Akt\_inactive]) - V_{\text{"Neuroblastoma Cell Cytoplasm"}} \cdot \text{HMM\_Modified}(Kcat_{(Akt\_Activation\_TCL1)}, km_{(Akt\_Activation\_TCL1)}, [TCL1], [Akt\_inactive])$$

$$\frac{d([C3G\_inactive] \cdot V_{\text{"Neuroblastoma Cell Cytoplasm"}})}{dt} = +V_{\text{"Neuroblastoma Cell Cytoplasm"}} \cdot k1_{(C3G\_Deactivation)} \cdot [C3G\_active] - V_{\text{"Neuroblastoma Cell Cytoplasm"}} \cdot \text{HMM\_Modified}(KcatC3GNGF, KmC3GNGF, [NGFR\_bound], [C3G\_inactive])$$

$$\frac{d([Raf1\_inactive] \cdot V_{\text{"Neuroblastoma Cell Cytoplasm"}})}{dt} = +V_{\text{"Neuroblastoma Cell Cytoplasm"}} \cdot \text{HMM\_Modified}(Kcat_{(Raf1\_Deactivation\_by\_Akt)}, km_{(Raf1\_Deactivation\_by\_Akt)}, [Akt\_active], [Raf1\_active]) - V_{\text{"Neuroblastoma Cell Cytoplasm"}} \cdot \text{HMM\_Modified}(Kcat_{(\text{"Raf1 activation\_by\_Ras"})}, km_{(\text{"Raf1 activation\_by\_Ras"})}, [RAS\_active], [Raf1\_inactive]) + V_{\text{"Neuroblastoma Cell Cytoplasm"}} \cdot \text{HMM\_Modified}(Kcat_{(Raf1\_deactivation\_by\_ERK)}, km_{(Raf1\_deactivation\_by\_ERK)}, [ERK\_active], [Raf1\_active]) + V_{\text{"Neuroblastoma Cell Cytoplasm"}} \cdot \text{HMM\_Modified}(Kcat_{(Raf1\_Feedback\_Deactivation\_Raf1PPtase)}, km_{(Raf1\_Feedback\_Deactivation\_Raf1PPtase)}, [Raf1PPtase], [Raf1\_active])$$

$$\frac{d([degradedEGFR] \cdot V_{\text{"Neuroblastoma Cell Cytoplasm"}})}{dt} = +V_{\text{"Neuroblastoma Cell Cytoplasm"}} \cdot k1_{(\text{"EGFR\_free degradation"})} \cdot [EGFR\_free] + V_{\text{"Neuroblastoma Cell Cytoplasm"}} \cdot k1_{(EGFR\_bound\_degradation)} \cdot [EGFR\_bound]$$

$$\frac{d([\text{Akt\_active}]\cdot V_{\text{"Neuroblastoma Cell Cytoplasm"}})}{dt} = -V_{\text{"Neuroblastoma Cell Cytoplasm"}}\cdot \text{HMM\_Modified}(Kcat_{(\text{Akt\_Deactivation\_PP2A})}, km_{(\text{Akt\_Deactivation\_PP2A})}, [\text{PP2A\_active}], [\text{Akt\_active}])$$
$$-V_{\text{"Neuroblastoma Cell Cytoplasm"}}\cdot \text{HMM\_Modified}(Kcat_{(\text{Akt\_Deactivation\_PHLPP})}, km_{(\text{Akt\_Deactivation\_PHLPP})}, [\text{PHLPP}], [\text{Akt\_active}])$$
$$-V_{\text{"Neuroblastoma Cell Cytoplasm"}}\cdot \text{HMM\_Modified}(Kcat_{(\text{Akt\_Deactivation\_CTMP})}, km_{(\text{Akt\_Deactivation\_CTMP})}, [\text{CTMP}], [\text{Akt\_active}])$$
$$+V_{\text{"Neuroblastoma Cell Cytoplasm"}}\cdot \text{HMM\_Modified}(Kcat_{(\text{Akt\_Activation\_mTORC2})}, km_{(\text{Akt\_Activation\_mTORC2})}, [\text{mTORC2\_active}], [\text{Akt\_Thr308}])$$
$$+V_{\text{"Neuroblastoma Cell Cytoplasm"}}\cdot \text{HMM\_Modified}(Kcat_{(\text{"Akt\_Activation\_HSP90-Cdc37"})}, km_{(\text{"Akt\_Activation\_HSP90-Cdc37"})}, [\text{"HSP90-Cdc37Active"}], [\text{Akt\_inactive}])$$
$$+V_{\text{"Neuroblastoma Cell Cytoplasm"}}\cdot \text{HMM\_Modified}(Kcat_{(\text{Akt\_Activation\_TCL1})}, km_{(\text{Akt\_Activation\_TCL1})}, [\text{TCL1}], [\text{Akt\_inactive}])$$

$$\frac{d([\text{C3G\_active}]\cdot V_{\text{"Neuroblastoma Cell Cytoplasm"}})}{dt} = -V_{\text{"Neuroblastoma Cell Cytoplasm"}}\cdot k1_{(\text{C3G\_Deactivation})}\cdot [\text{C3G\_active}]$$
$$+V_{\text{"Neuroblastoma Cell Cytoplasm"}}\cdot \text{HMM\_Modified}(\text{KcatC3GNGF}, \text{KmC3GNGF}, [\text{NGFR\_bound}], [\text{C3G\_inactive}])$$

$$\frac{d([\text{Rap1\_active}]\cdot V_{\text{"Neuroblastoma Cell Cytoplasm"}})}{dt} = +V_{\text{"Neuroblastoma Cell Cytoplasm"}}\cdot \text{HMM\_Modified}(\text{KcatC3G}, \text{KmC3G}, [\text{C3G\_active}], [\text{Rap1\_inactive}])$$
$$-V_{\text{"Neuroblastoma Cell Cytoplasm"}}\cdot \text{HMM\_Modified}(Kcat_{(\text{Rap1\_Feedback\_Deactivation\_Rap1Gap})}, km_{(\text{Rap1\_Feedback\_Deactivation\_Rap1Gap})}, [\text{Rap1Gap}], [\text{Rap1\_active}])$$

$$\frac{d([\text{Rap1\_inactive}]\cdot V_{\text{"Neuroblastoma Cell Cytoplasm"}})}{dt} = -V_{\text{"Neuroblastoma Cell Cytoplasm"}}\cdot \text{HMM\_Modified}(\text{KcatC3G}, \text{KmC3G}, [\text{C3G\_active}], [\text{Rap1\_inactive}])$$
$$+V_{\text{"Neuroblastoma Cell Cytoplasm"}}\cdot \text{HMM\_Modified}(Kcat_{(\text{Rap1\_Feedback\_Deactivation\_Rap1Gap})}, km_{(\text{Rap1\_Feedback\_Deactivation\_Rap1Gap})}, [\text{Rap1Gap}], [\text{Rap1\_active}])$$

$$\frac{d([\text{bRaf\_active}]\cdot V_{\text{"Neuroblastoma Cell Cytoplasm"}})}{dt} = -V_{\text{"Neuroblastoma Cell Cytoplasm"}}\cdot \text{HMM\_Modified}(Kcat_{(\text{bRaf\_Deactivation\_Raf1PPtase})}, km_{(\text{bRaf\_Deactivation\_Raf1PPtase})}, [\text{Raf1PPtase}], [\text{bRaf\_active}])$$
$$+V_{\text{"Neuroblastoma Cell Cytoplasm"}}\cdot \text{HMM\_Modified}(\text{KcatRap1toBRaf}, \text{KmRap1toBraf}, [\text{Rap1\_active}], [\text{bRaf\_inactive}])$$
$$+V_{\text{"Neuroblastoma Cell Cytoplasm"}}\cdot \text{HMM\_Modified}(Kcat_{(\text{bRAF\_activation\_RAS})}, km_{(\text{bRAF\_activation\_RAS})}, [\text{RAS\_active}], [\text{bRaf\_inactive}])$$

$$\frac{d([\text{PP2A\_active}]\cdot V_{\text{"Neuroblastoma Cell Cytoplasm"}})}{dt} = +\text{HMM\_Modified}(Kcat_{(\text{PP2A\_activation\_by\_p38})}, km_{(\text{PP2A\_activation\_by\_p38})}, [\text{p38\_active}], [\text{PP2A\_inactive}])$$

$$\frac{d([\text{Mek\_active}]\cdot V_{\text{"Neuroblastoma Cell Cytoplasm"}})}{dt} = +V_{\text{"Neuroblastoma Cell Cytoplasm"}}\cdot \text{HMM\_Modified}(Kcat_{(\text{Mek\_Activation\_Raf1})}, km_{(\text{Mek\_Activation\_Raf1})}, [\text{Raf1\_active}], [\text{Mek\_inactive}])$$
$$-V_{\text{"Neuroblastoma Cell Cytoplasm"}}\cdot \text{HMM\_Modified}(Kcat_{(\text{Mek\_Feedback\_Deactivation\_PP2A})}, km_{(\text{Mek\_Feedback\_Deactivation\_PP2A})}, [\text{PP2A\_active}], [\text{Mek\_active}])$$
$$+V_{\text{"Neuroblastoma Cell Cytoplasm"}}\cdot \text{HMM\_Modified}(Kcat_{(\text{Mek\_Activation\_bRaf})}, km_{(\text{Mek\_Activation\_bRaf})}, [\text{bRaf\_active}], [\text{Mek\_inactive}])$$

$$\frac{d\left([\text{bRaf\_inactive}]\cdot V_{\text{"Neuroblastoma Cell Cytoplasm"}}\right)}{dt} = +V_{\text{"Neuroblastoma Cell Cytoplasm"}}\cdot \text{HMM\_Modified}\left(\text{Kcat}_{(\text{bRaf\_Deactivation\_Raf1PPtase})}, \text{km}_{(\text{bRaf\_Deactivation\_Raf1PPtase})}, [\text{Raf1PPtase}], [\text{bRaf\_active}]\right)$$
$$-V_{\text{"Neuroblastoma Cell Cytoplasm"}}\cdot \text{HMM\_Modified}\left(\text{KcatRap1toBRaf}, \text{KmRap1toBraf}, [\text{Rap1\_active}], [\text{bRaf\_inactive}]\right)$$
$$-V_{\text{"Neuroblastoma Cell Cytoplasm"}}\cdot \text{HMM\_Modified}\left(\text{Kcat}_{(\text{bRAF\_activation\_RAS})}, \text{km}_{(\text{bRAF\_activation\_RAS})}, [\text{RAS\_active}], [\text{bRaf\_inactive}]\right)$$

$$\frac{d\left([\text{ERK\_inactive}]\cdot V_{\text{"Neuroblastoma Cell Cytoplasm"}}\right)}{dt} = -V_{\text{"Neuroblastoma Cell Cytoplasm"}}\cdot \text{HMM\_Modified}\left(\text{Kcat}_{(\text{Erk\_Activation})}, \text{km}_{(\text{Erk\_Activation})}, [\text{Mek\_active}], [\text{ERK\_inactive}]\right)$$
$$+V_{\text{"Neuroblastoma Cell Cytoplasm"}}\cdot \text{HMM\_Modified}\left(\text{Kcat}_{(\text{Erk\_Feedback\_Deactivation\_PP2A})}, \text{km}_{(\text{Erk\_Feedback\_Deactivation\_PP2A})}, [\text{PP2A\_active}], [\text{ERK\_active}]\right)$$
$$+V_{\text{"Neuroblastoma Cell Cytoplasm"}}\cdot \text{HMM\_Modified}\left(\text{Kcat}_{(\text{Erk\_Feedback\_Deactivation\_Raf1})}, \text{km}_{(\text{Erk\_Feedback\_Deactivation\_Raf1})}, [\text{Raf1\_active}], [\text{ERK\_active}]\right)$$
$$+\text{HMM\_Modified}\left(\text{Kcat}_{(\text{ERK\_inhibition\_by\_DUSP2})}, \text{km}_{(\text{ERK\_inhibition\_by\_DUSP2})}, [\text{DUSP2\_active}], [\text{ERK\_active}]\right)$$
$$-V_{\text{"Neuroblastoma Cell Cytoplasm"}}\cdot \text{"HMM modified"}\left(\text{Kcat}_{(\text{ERK\_activation\_by\_ALK})}, [\text{ALK\_active}], [\text{ERK\_inactive}], \text{km}_{(\text{ERK\_activation\_by\_ALK})}\right)$$

$$\frac{d\left([\text{PI3K\_active}]\cdot V_{\text{"Neuroblastoma Cell Cytoplasm"}}\right)}{dt} = -V_{\text{"Neuroblastoma Cell Cytoplasm"}}\cdot \text{k1}_{(\text{PI3K\_Deactivation})}\cdot[\text{PI3K\_active}]$$
$$+V_{\text{"Neuroblastoma Cell Cytoplasm"}}\cdot \text{HMM\_Modified}\left(\text{Kcat}_{(\text{PI3K\_Activation\_IRS1})}, \text{km}_{(\text{PI3K\_Activation\_IRS1})}, [\text{IRS1\_active}], [\text{PI3K\_inactive}]\right)$$
$$+V_{\text{"Neuroblastoma Cell Cytoplasm"}}\cdot \text{HMM\_Modified}\left(\text{Kcat}_{(\text{PI3K\_Activation\_Ras})}, \text{km}_{(\text{PI3K\_Activation\_Ras})}, [\text{RAS\_active}], [\text{PI3K\_inactive}]\right)$$
$$+V_{\text{"Neuroblastoma Cell Cytoplasm"}}\cdot \text{HMM\_Modified}\left(\text{Kcat}_{(\text{PI3K\_Activation\_EGFR\_di\_p})}, \text{km}_{(\text{PI3K\_Activation\_EGFR\_di\_p})}, [\text{EGFR\_bound}], [\text{PI3K\_inactive}]\right)$$
$$+V_{\text{"Neuroblastoma Cell Cytoplasm"}}\cdot \text{HMM\_Modified}\left(\text{Kcat}_{(\text{PI3K\_activation\_by\_ALK})}, \text{km}_{(\text{PI3K\_activation\_by\_ALK})}, [\text{ALK\_active}], [\text{PI3K\_inactive}]\right)$$

$$\frac{d\left([\text{PI3K\_inactive}]\cdot V_{\text{"Neuroblastoma Cell Cytoplasm"}}\right)}{dt} = +V_{\text{"Neuroblastoma Cell Cytoplasm"}}\cdot \text{k1}_{(\text{PI3K\_Deactivation})}\cdot[\text{PI3K\_active}]$$
$$-V_{\text{"Neuroblastoma Cell Cytoplasm"}}\cdot \text{HMM\_Modified}\left(\text{Kcat}_{(\text{PI3K\_Activation\_IRS1})}, \text{km}_{(\text{PI3K\_Activation\_IRS1})}, [\text{IRS1\_active}], [\text{PI3K\_inactive}]\right)$$
$$-V_{\text{"Neuroblastoma Cell Cytoplasm"}}\cdot \text{HMM\_Modified}\left(\text{Kcat}_{(\text{PI3K\_Activation\_Ras})}, \text{km}_{(\text{PI3K\_Activation\_Ras})}, [\text{RAS\_active}], [\text{PI3K\_inactive}]\right)$$
$$-V_{\text{"Neuroblastoma Cell Cytoplasm"}}\cdot \text{HMM\_Modified}\left(\text{Kcat}_{(\text{PI3K\_Activation\_EGFR\_di\_p})}, \text{km}_{(\text{PI3K\_Activation\_EGFR\_di\_p})}, [\text{EGFR\_bound}], [\text{PI3K\_inactive}]\right)$$
$$-V_{\text{"Neuroblastoma Cell Cytoplasm"}}\cdot \text{HMM\_Modified}\left(\text{Kcat}_{(\text{PI3K\_activation\_by\_ALK})}, \text{km}_{(\text{PI3K\_activation\_by\_ALK})}, [\text{ALK\_active}], [\text{PI3K\_inactive}]\right)$$

$$
\begin{aligned}
\frac{d([\text{ERK\_active}]\cdot V_{\text{"Neuroblastoma Cell Cytoplasm"}})}{dt} =\ & +V_{\text{"Neuroblastoma Cell Cytoplasm"}}\cdot \text{HMM\_Modified}(Kcat_{(\text{Erk\_Activation})}, km_{(\text{Erk\_Activation})}, [\text{Mek\_active}], [\text{ERK\_inactive}]) \\
& -V_{\text{"Neuroblastoma Cell Cytoplasm"}}\cdot \text{HMM\_Modified}(Kcat_{(\text{Erk\_Feedback\_Deactivation\_PP2A})}, km_{(\text{Erk\_Feedback\_Deactivation\_PP2A})}, [\text{PP2A\_active}], [\text{ERK\_active}]) \\
& -V_{\text{"Neuroblastoma Cell Cytoplasm"}}\cdot \text{HMM\_Modified}(Kcat_{(\text{Erk\_Feedback\_Deactivation\_Raf1})}, km_{(\text{Erk\_Feedback\_Deactivation\_Raf1})}, [\text{Raf1\_active}], [\text{ERK\_active}]) \\
& -\text{HMM\_Modified}(Kcat_{(\text{ERK\_inhibition\_by\_DUSP2})}, km_{(\text{ERK\_inhibition\_by\_DUSP2})}, [\text{DUSP2\_active}], [\text{ERK\_active}]) \\
& +V_{\text{"Neuroblastoma Cell Cytoplasm"}}\cdot \text{"HMM modified"}(Kcat_{(\text{ERK\_activation\_by\_ALK})}, [\text{ALK\_active}], [\text{ERK\_inactive}], km_{(\text{ERK\_activation\_by\_ALK})})
\end{aligned}
$$

$$
\begin{aligned}
\frac{d([\text{Raf1\_active}]\cdot V_{\text{"Neuroblastoma Cell Cytoplasm"}})}{dt} =\ & -V_{\text{"Neuroblastoma Cell Cytoplasm"}}\cdot \text{HMM\_Modified}(Kcat_{(\text{Raf1\_Deactivation\_by\_Akt})}, km_{(\text{Raf1\_Deactivation\_by\_Akt})}, [\text{Akt\_active}], [\text{Raf1\_active}]) \\
& +V_{\text{"Neuroblastoma Cell Cytoplasm"}}\cdot \text{HMM\_Modified}(Kcat_{(\text{"Raf1 activation\_by\_Ras"})}, km_{(\text{"Raf1 activation\_by\_Ras"})}, [\text{RAS\_active}], [\text{Raf1\_inactive}]) \\
& -V_{\text{"Neuroblastoma Cell Cytoplasm"}}\cdot \text{HMM\_Modified}(Kcat_{(\text{Raf1\_deactivation\_by\_ERK})}, km_{(\text{Raf1\_deactivation\_by\_ERK})}, [\text{ERK\_active}], [\text{Raf1\_active}]) \\
& -V_{\text{"Neuroblastoma Cell Cytoplasm"}}\cdot \text{HMM\_Modified}(Kcat_{(\text{Raf1\_Feedback\_Deactivation\_Raf1PPtase})}, km_{(\text{Raf1\_Feedback\_Deactivation\_Raf1PPtase})}, [\text{Raf1PPtase}], [\text{Raf1\_active}])
\end{aligned}
$$

$$
\begin{aligned}
\frac{d([\text{Mek\_inactive}]\cdot V_{\text{"Neuroblastoma Cell Cytoplasm"}})}{dt} =\ & -V_{\text{"Neuroblastoma Cell Cytoplasm"}}\cdot \text{HMM\_Modified}(Kcat_{(\text{Mek\_Activation\_Raf1})}, km_{(\text{Mek\_Activation\_Raf1})}, [\text{Raf1\_active}], [\text{Mek\_inactive}]) \\
& +V_{\text{"Neuroblastoma Cell Cytoplasm"}}\cdot \text{HMM\_Modified}(Kcat_{(\text{Mek\_Feedback\_Deactivation\_PP2A})}, km_{(\text{Mek\_Feedback\_Deactivation\_PP2A})}, [\text{PP2A\_active}], [\text{Mek\_active}]) \\
& -V_{\text{"Neuroblastoma Cell Cytoplasm"}}\cdot \text{HMM\_Modified}(Kcat_{(\text{Mek\_Activation\_bRaf})}, km_{(\text{Mek\_Activation\_bRaf})}, [\text{bRaf\_active}], [\text{Mek\_inactive}])
\end{aligned}
$$

$$
\begin{aligned}
\frac{d([\text{degradedNGFR}]\cdot V_{\text{"Neuroblastoma Cell Cytoplasm"}})}{dt} =\ & +V_{\text{"Neuroblastoma Cell Cytoplasm"}}\cdot k1_{(\text{"NGFR degradation"})}\cdot[\text{NGFR}] \\
& +V_{\text{"Neuroblastoma Cell Cytoplasm"}}\cdot k1_{(\text{"bNGFR degradation"})}\cdot[\text{NGFR\_bound}]
\end{aligned}
$$

$$
\frac{d([\text{STAT1}]\cdot V_{\text{"Neuroblastoma Cell Nucleus"}})}{dt} = -\text{"HMM\_activation and inhibition\_STAT1"}(Kcat_{(\text{STAT1\_activation})}, [\text{EGFR\_bound}], [\text{PIAS3}], Ki1_{(\text{STAT1\_activation})}, [\text{SOCS1}], Ki2_{(\text{STAT1\_activation})}, [\text{SOCS3}], Ki3_{(\text{STAT1\_activation})}, [\text{STAT1}], Km_{(\text{STAT1\_activation})})
$$

$$
\begin{aligned}
\frac{d([\text{STAT1\_p}]\cdot V_{\text{"Neuroblastoma Cell Nucleus"}})}{dt} =\ & +\text{"HMM\_activation and inhibition\_STAT1"}(Kcat_{(\text{STAT1\_activation})}, [\text{EGFR\_bound}], [\text{PIAS3}], Ki1_{(\text{STAT1\_activation})}, [\text{SOCS1}], Ki2_{(\text{STAT1\_activation})}, [\text{SOCS3}], Ki3_{(\text{STAT1\_activation})}, [\text{STAT1}], Km_{(\text{STAT1\_activation})}) \\
& -V_{\text{"Neuroblastoma Cell Nucleus"}}\cdot \text{"Henri-Michaelis-Menten (irreversible)"}([\text{STAT1\_p}], Km_{(\text{PDL1\_transcrition\_by\_STAT1\_p})}, V_{(\text{PDL1\_transcrition\_by\_STAT1\_p})})
\end{aligned}
$$

$$
\begin{aligned}
\frac{d([\text{STAT3}]\cdot V_{\text{"Neuroblastoma Cell Nucleus"}})}{dt} =\ & -V_{\text{"Neuroblastoma Cell Nucleus"}}\cdot \text{HMM\_Modified}(Kcat_{(\text{STAT3\_acetilation})}, km_{(\text{STAT3\_acetilation})}, [\text{CREBBP}], [\text{STAT3}]) \\
& -\text{"HMM modified"}(Kcat_{(\text{STAT3\_phosporilation\_by\_mTORC1})}, [\text{mTORC1\_active}], [\text{STAT3}], km_{(\text{STAT3\_phosporilation\_by\_mTORC1})}) \\
& -\text{HMM\_Modified}(Kcat_{(\text{STAT3\_phosphorilation\_by\_ALK})}, km_{(\text{STAT3\_phosphorilation\_by\_ALK})}, [\text{ALK\_active}], [\text{STAT3}])
\end{aligned}
$$

[illegible]

$$\frac{d\left([\text{JNK\_active}]\cdot V_{\text{"Neuroblastoma Cell Cytoplasm"}}\right)}{dt} = -\text{HMM\_Modified}\left(\text{Kcat}_{(\text{JNK\_inhibition\_by\_DUSP4/6})}, \text{km}_{(\text{JNK\_inhibition\_by\_DUSP4/6})}, [\text{DUSP4/6\_active}], [\text{JNK\_active}]\right) + V_{\text{"Neuroblastoma Cell Cytoplasm"}}\cdot\text{HMM\_Modified}\left(\text{Kcat}_{(\text{JNK\_activation\_by\_MKK4/7})}, \text{km}_{(\text{JNK\_activation\_by\_MKK4/7})}, [\text{MKK4/7\_active}], [\text{JNK\_inactive}]\right)$$

$$\frac{d\left([\text{MKK3/6\_active}]\cdot V_{\text{"Neuroblastoma Cell Cytoplasm"}}\right)}{dt} = +V_{\text{"Neuroblastoma Cell Cytoplasm"}}\cdot\text{"Henri-Michaelis-Menten (irreversible)"}\left([\text{MKK3/6\_inactive}], \text{Km}_{(\text{MKK3/6\_activation})}, V_{(\text{MKK3/6\_activation})}\right)$$

$$\frac{d\left([\text{MKK3/6\_inactive}]\cdot V_{\text{"Neuroblastoma Cell Cytoplasm"}}\right)}{dt} = -V_{\text{"Neuroblastoma Cell Cytoplasm"}}\cdot\text{"Henri-Michaelis-Menten (irreversible)"}\left([\text{MKK3/6\_inactive}], \text{Km}_{(\text{MKK3/6\_activation})}, V_{(\text{MKK3/6\_activation})}\right)$$

$$\frac{d\left([\text{p38\_active}]\cdot V_{\text{"Neuroblastoma Cell Nucleus"}}\right)}{dt} = +\text{HMM\_Modified}\left(\text{Kcat}_{(\text{p38\_activation})}, \text{km}_{(\text{p38\_activation})}, [\text{MKK3/6\_active}], [\text{p38\_inactive}]\right) - V_{\text{"Neuroblastoma Cell Nucleus"}}\cdot\text{HMM\_Modified}\left(\text{Kcat}_{(\text{p38\_inhibition\_by\_DUSP2})}, \text{km}_{(\text{p38\_inhibition\_by\_DUSP2})}, [\text{DUSP2\_active}], [\text{p38\_active}]\right)$$

$$\frac{d\left([\text{p38\_inactive}]\cdot V_{\text{"Neuroblastoma Cell Nucleus"}}\right)}{dt} = -\text{HMM\_Modified}\left(\text{Kcat}_{(\text{p38\_activation})}, \text{km}_{(\text{p38\_activation})}, [\text{MKK3/6\_active}], [\text{p38\_inactive}]\right) + V_{\text{"Neuroblastoma Cell Nucleus"}}\cdot\text{HMM\_Modified}\left(\text{Kcat}_{(\text{p38\_inhibition\_by\_DUSP2})}, \text{km}_{(\text{p38\_inhibition\_by\_DUSP2})}, [\text{DUSP2\_active}], [\text{p38\_active}]\right)$$

$$\frac{d\left([\text{PP2A\_inactive}]\cdot V_{\text{"Neuroblastoma Cell Cytoplasm"}}\right)}{dt} = -\text{HMM\_Modified}\left(\text{Kcat}_{(\text{PP2A\_activation\_by\_p38})}, \text{km}_{(\text{PP2A\_activation\_by\_p38})}, [\text{p38\_active}], [\text{PP2A\_inactive}]\right)$$

$$\frac{d\left([\text{DUSP4/6\_active}]\cdot V_{\text{"Neuroblastoma Cell Nucleus"}}\right)}{dt} = +\text{HMM\_Modified}\left(\text{Kcat}_{(\text{DUSP4/6\_activation\_by\_ERK})}, \text{km}_{(\text{DUSP4/6\_activation\_by\_ERK})}, [\text{ERK\_active}], [\text{DUSP4/6\_inactive}]\right)$$

$$\frac{d\left([\text{DUSP4/6\_inactive}]\cdot V_{\text{"Neuroblastoma Cell Nucleus"}}\right)}{dt} = -\text{HMM\_Modified}\left(\text{Kcat}_{(\text{DUSP4/6\_activation\_by\_ERK})}, \text{km}_{(\text{DUSP4/6\_activation\_by\_ERK})}, [\text{ERK\_active}], [\text{DUSP4/6\_inactive}]\right)$$

$$\frac{d\left([\text{JNK\_inactive}]\cdot V_{\text{"Neuroblastoma Cell Cytoplasm"}}\right)}{dt} = +\text{HMM\_Modified}\left(\text{Kcat}_{(\text{JNK\_inhibition\_by\_DUSP4/6})}, \text{km}_{(\text{JNK\_inhibition\_by\_DUSP4/6})}, [\text{DUSP4/6\_active}], [\text{JNK\_active}]\right) - V_{\text{"Neuroblastoma Cell Cytoplasm"}}\cdot\text{HMM\_Modified}\left(\text{Kcat}_{(\text{JNK\_activation\_by\_MKK4/7})}, \text{km}_{(\text{JNK\_activation\_by\_MKK4/7})}, [\text{MKK4/7\_active}], [\text{JNK\_inactive}]\right)$$

$$\frac{d\left([\text{MKK4/7\_active}]\cdot V_{\text{"Neuroblastoma Cell Cytoplasm"}}\right)}{dt} = +V_{\text{"Neuroblastoma Cell Cytoplasm"}}\cdot\text{HMM\_Modified}\left(\text{Kcat}_{(\text{MKK4/7\_activation})}, \text{km}_{(\text{MKK4/7\_activation})}, [\text{ASK/MLK\_active}], [\text{MKK4/7\_inactive}]\right) - V_{\text{"Neuroblastoma Cell Cytoplasm"}}\cdot\text{HMM\_Modified}\left(\text{Kcat}_{(\text{MKK4/7\_inhibition\_by\_Akt})}, \text{km}_{(\text{MKK4/7\_inhibition\_by\_Akt})}, [\text{Akt\_active}], [\text{MKK4/7\_active}]\right)$$

$$\frac{d\left([\text{MKK4/7\_inactive}]\cdot V_{\text{"Neuroblastoma Cell Cytoplasm"}}\right)}{dt} = -V_{\text{"Neuroblastoma Cell Cytoplasm"}}\cdot\text{HMM\_Modified}\left(\text{Kcat}_{(\text{MKK4/7\_activation})}, \text{km}_{(\text{MKK4/7\_activation})}, [\text{ASK/MLK\_active}], [\text{MKK4/7\_inactive}]\right) + V_{\text{"Neuroblastoma Cell Cytoplasm"}}\cdot\text{HMM\_Modified}\left(\text{Kcat}_{(\text{MKK4/7\_inhibition\_by\_Akt})}, \text{km}_{(\text{MKK4/7\_inhibition\_by\_Akt})}, [\text{Akt\_active}], [\text{MKK4/7\_active}]\right)$$

$$\frac{d\left([\text{DUSP2\_active}]\cdot V_{\text{"Neuroblastoma Cell Nucleus"}}\right)}{dt} = +\text{HMM\_Modified}\left(\text{Kcat}_{(\text{DUSP2\_transcription\_by\_JNK})}, \text{km}_{(\text{DUSP2\_transcription\_by\_JNK})}, [\text{JNK\_active}], [\text{DUSP2\_inactive}]\right) + \text{"HMM modified"}\left(\text{Kcat}_{(\text{"DUSP2\_transcription\_by\_N-MYC"})}, [\text{"N-MYC"}], [\text{DUSP2\_inactive}], \text{km}_{(\text{"DUSP2\_transcription\_by\_N-MYC"})}\right)$$

$$\frac{d\left([\text{DUSP2\_inactive}]\cdot V_{\text{"Neuroblastoma Cell Nucleus"}}\right)}{dt} = -\text{HMM\_Modified}\left(\text{Kcat}_{(\text{DUSP2\_transcription\_by\_JNK})}, \text{km}_{(\text{DUSP2\_transcription\_by\_JNK})}, [\text{JNK\_active}], [\text{DUSP2\_inactive}]\right) - \text{"HMM modified"}\left(\text{Kcat}_{(\text{"DUSP2\_transcription\_by\_N-MYC"})}, [\text{"N-MYC"}], [\text{DUSP2\_inactive}], \text{km}_{(\text{"DUSP2\_transcription\_by\_N-MYC"})}\right)$$

$$\frac{d\left([\text{MYCN\_mRNA}]\cdot V_{\text{"Neuroblastoma Cell Nucleus"}}\right)}{dt} = +\text{HMM\_Modified}\left(\text{Kcat}_{(\text{MYCN\_transcription\_by\_STAT3\_p})}, \text{km}_{(\text{MYCN\_transcription\_by\_STAT3\_p})}, [\text{STAT3\_p}], [\text{MYCN}]\right) - V_{\text{"Neuroblastoma Cell Nucleus"}}\cdot \text{k1}_{(\text{MYCN\_mRNA\_degradation})}\cdot[\text{MYCN\_mRNA}] - \text{"Henri-Michaelis-Menten (irreversible)"}\left([\text{MYCN\_mRNA}], \text{Km}_{(\text{"N-MYC\_translation\_by\_MYCN\_mRNA"})}, V_{(\text{"N-MYC\_translation\_by\_MYCN\_mRNA"})}\right)$$

$$\frac{d\left([\text{GSK3b\_active}]\cdot V_{\text{"Neuroblastoma Cell Cytoplasm"}}\right)}{dt} = -V_{\text{"Neuroblastoma Cell Cytoplasm"}}\cdot \text{HMM\_Modified}\left(\text{Kcat}_{(\text{GSK3b\_inactivation\_by\_Akt})}, \text{km}_{(\text{GSK3b\_inactivation\_by\_Akt})}, [\text{Akt\_active}], [\text{GSK3b\_active}]\right)$$

$$\frac{d\left([\text{GSK3b\_inactive}]\cdot V_{\text{"Neuroblastoma Cell Cytoplasm"}}\right)}{dt} = +V_{\text{"Neuroblastoma Cell Cytoplasm"}}\cdot \text{HMM\_Modified}\left(\text{Kcat}_{(\text{GSK3b\_inactivation\_by\_Akt})}, \text{km}_{(\text{GSK3b\_inactivation\_by\_Akt})}, [\text{Akt\_active}], [\text{GSK3b\_active}]\right)$$

$$\frac{d\left([\text{"N-MYC"}]\cdot V_{\text{"Neuroblastoma Cell Cytoplasm"}}\right)}{dt} = -V_{\text{"Neuroblastoma Cell Cytoplasm"}}\cdot \text{HMM\_Modified}\left(\text{Kcat}_{(\text{"N-MYC\_degradation\_by\_GSK3b"})}, \text{km}_{(\text{"N-MYC\_degradation\_by\_GSK3b"})}, [\text{GSK3b\_active}], [\text{"N-MYC"}]\right) + \text{"Henri-Michaelis-Menten (irreversible)"}\left([\text{MYCN\_mRNA}], \text{Km}_{(\text{"N-MYC\_translation\_by\_MYCN\_mRNA"})}, V_{(\text{"N-MYC\_translation\_by\_MYCN\_mRNA"})}\right)$$

$$\frac{d\left([\text{MYCN\_mRNA\_degraded}]\cdot V_{\text{"Neuroblastoma Cell Nucleus"}}\right)}{dt} = +V_{\text{"Neuroblastoma Cell Nucleus"}}\cdot \text{k1}_{(\text{MYCN\_mRNA\_degradation})}\cdot[\text{MYCN\_mRNA}]$$

$$\frac{d\left([\text{"N-MYC\_pp"}]\cdot V_{\text{"Neuroblastoma Cell Cytoplasm"}}\right)}{dt} = +V_{\text{"Neuroblastoma Cell Cytoplasm"}}\cdot \text{HMM\_Modified}\left(\text{Kcat}_{(\text{"N-MYC\_degradation\_by\_GSK3b"})}, \text{km}_{(\text{"N-MYC\_degradation\_by\_GSK3b"})}, [\text{GSK3b\_active}], [\text{"N-MYC"}]\right)$$

$$\frac{d\left([\text{ALK\_active}]\cdot V_{\text{"Neuroblastoma Cell Cytoplasm"}}\right)}{dt} = +V_{\text{"Neuroblastoma Cell Cytoplasm"}}\cdot\left(\text{k1}_{(\text{ALK\_activation})}\cdot[\text{ALK\_inactive}] - \text{k2}_{(\text{ALK\_activation})}\cdot[\text{ALK\_active}]\right)$$

$$\frac{d\left([\text{Akt\_Thr308}]\cdot V_{\text{"Neuroblastoma Cell Cytoplasm"}}\right)}{dt} = -V_{\text{"Neuroblastoma Cell Cytoplasm"}}\cdot \text{HMM\_Modified}\left(\text{Kcat}_{(\text{Akt\_Activation\_mTORC2})}, \text{km}_{(\text{Akt\_Activation\_mTORC2})}, [\text{mTORC2\_active}], [\text{Akt\_Thr308}]\right) + V_{\text{"Neuroblastoma Cell Cytoplasm"}}\cdot \text{HMM\_Modified}\left(\text{Kcat}_{(\text{Akt\_Activation\_PDK1})}, \text{km}_{(\text{Akt\_Activation\_PDK1})}, [\text{PDK1\_active}], [\text{Akt\_conformational\_change}]\right)$$

[illegible]

$$\frac{d([Akt\_conformational\_change] \cdot V_{\text{"Neuroblastoma Cell Cytoplasm"}})}{dt} = +V_{\text{"Neuroblastoma Cell Cytoplasm"}} \cdot HMM\_Modified(Kcat_{(Akt\_Activation\_PIP3)}, km_{(Akt\_Activation\_PIP3)}, [PIP3], [Akt\_inactive]) - V_{\text{"Neuroblastoma Cell Cytoplasm"}} \cdot HMM\_Modified(Kcat_{(Akt\_Activation\_PDK1)}, km_{(Akt\_Activation\_PDK1)}, [PDK1\_active], [Akt\_conformational\_change])$$

$$\frac{d([PIP3] \cdot V_{\text{"Neuroblastoma Cell Cytoplasm"}})}{dt} = +V_{\text{"Neuroblastoma Cell Cytoplasm"}} \cdot HMM\_Modified(Kcat_{(PIP3\_Activation)}, km_{(PIP3\_Activation)}, [PI3K\_active], [PIP2]) - V_{\text{"Neuroblastoma Cell Cytoplasm"}} \cdot HMM\_Modified(Kcat_{(PIP3\_Feedback\_Deactivation\_PTEN)}, km_{(PIP3\_Feedback\_Deactivation\_PTEN)}, [PTEN\_active], [PIP3])$$

$$\frac{d([PIP2] \cdot V_{\text{"Neuroblastoma Cell Cytoplasm"}})}{dt} = -V_{\text{"Neuroblastoma Cell Cytoplasm"}} \cdot HMM\_Modified(Kcat_{(PIP3\_Activation)}, km_{(PIP3\_Activation)}, [PI3K\_active], [PIP2]) + V_{\text{"Neuroblastoma Cell Cytoplasm"}} \cdot HMM\_Modified(Kcat_{(PIP3\_Feedback\_Deactivation\_PTEN)}, km_{(PIP3\_Feedback\_Deactivation\_PTEN)}, [PTEN\_active], [PIP3])$$

$$\frac{d([ALK\_inactive] \cdot V_{\text{"Neuroblastoma Cell Cytoplasm"}})}{dt} = -V_{\text{"Neuroblastoma Cell Cytoplasm"}} \cdot (k1_{(ALK\_activation)} \cdot [ALK\_inactive] - k2_{(ALK\_activation)} \cdot [ALK\_active])$$

$$\frac{d([AMPK\_active] \cdot V_{\text{"Neuroblastoma Cell Cytoplasm"}})}{dt} = +V_{\text{"Neuroblastoma Cell Cytoplasm"}} \cdot (k1_{(AMPK\_activation)} \cdot [AMPK\_inactive] - k2_{(AMPK\_activation)} \cdot [AMPK\_active])$$

$$\frac{d([AMPK\_inactive] \cdot V_{\text{"Neuroblastoma Cell Cytoplasm"}})}{dt} = -V_{\text{"Neuroblastoma Cell Cytoplasm"}} \cdot (k1_{(AMPK\_activation)} \cdot [AMPK\_inactive] - k2_{(AMPK\_activation)} \cdot [AMPK\_active])$$

$$\frac{d([PTEN\_inactive] \cdot V_{\text{"Neuroblastoma Cell Cytoplasm"}})}{dt} = -V_{\text{"Neuroblastoma Cell Cytoplasm"}} \cdot (k1_{(PTEN\_activation)} \cdot [PTEN\_inactive] - k2_{(PTEN\_activation)} \cdot [PTEN\_active])$$

$$\frac{d\left([STAT3\_ac]\cdot V_{\text{"Neuroblastoma Cell Nucleus"}}\right)}{dt} = +V_{\text{"Neuroblastoma Cell Nucleus"}}\cdot \text{HMM\_Modified}\left(Kcat_{(STAT3\_acetylation)}, km_{(STAT3\_acetylation)}, [CREBBP], [STAT3]\right)$$

$$-\text{"HMM\_activation and inhibition\_STAT3"}\left(Kcat_{(STAT3\_phosphorylation\_by\_EGFR\_bound)}, [EGFR\_bound], [PIAS3], Ki1_{(STAT3\_phosphorylation\_by\_EGFR\_bound)}, [SOCS1], Ki2_{(STAT3\_phosphorylation\_by\_EGFR\_bound)}, [SOCS3], Ki3_{(STAT3\_phosphorylation\_by\_EGFR\_bound)}, [STAT3\_p], Ki4_{(STAT3\_phosphorylation\_by\_EGFR\_bound)}, [STAT3\_ac], Km_{(STAT3\_phosphorylation\_by\_EGFR\_bound)}\right)$$

$$\frac{d\left([STAT3\_ac\_p]\cdot V_{\text{"Neuroblastoma Cell Nucleus"}}\right)}{dt} = +\text{"HMM\_activation and inhibition\_STAT3"}\left(Kcat_{(STAT3\_phosphorylation\_by\_EGFR\_bound)}, [EGFR\_bound], [PIAS3], Ki1_{(STAT3\_phosphorylation\_by\_EGFR\_bound)}, [SOCS1], Ki2_{(STAT3\_phosphorylation\_by\_EGFR\_bound)}, [SOCS3], Ki3_{(STAT3\_phosphorylation\_by\_EGFR\_bound)}, [STAT3\_p], Ki4_{(STAT3\_phosphorylation\_by\_EGFR\_bound)}, [STAT3\_ac], Km_{(STAT3\_phosphorylation\_by\_EGFR\_bound)}\right)$$

$$-V_{\text{"Neuroblastoma Cell Nucleus"}}\cdot \text{"Henri-Michaelis-Menten (irreversible)"}\left([STAT3\_ac\_p], Km_{(PDL1\_transcription\_by\_STAT3\_ac\_p)}, V_{(PDL1\_transcription\_by\_STAT3\_ac\_p)}\right)$$

$$\frac{d\left([STAT3\_p]\cdot V_{\text{"Neuroblastoma Cell Nucleus"}}\right)}{dt} = +\text{"HMM modified"}\left(Kcat_{(STAT3\_phosphorylation\_by\_mTORC1)}, [mTORC1\_active], [STAT3], km_{(STAT3\_phosphorylation\_by\_mTORC1)}\right)$$

$$+\text{HMM\_Modified}\left(Kcat_{(STAT3\_phosphorylation\_by\_ALK)}, km_{(STAT3\_phosphorylation\_by\_ALK)}, [ALK\_active], [STAT3]\right)$$

$$-V_{\text{"Neuroblastoma Cell Nucleus"}}\cdot \text{"Henri-Michaelis-Menten (irreversible)"}\left([STAT3\_p], Km_{(PDL1\_transcription\_by\_STAT3\_p)}, V_{(PDL1\_transcription\_by\_STAT3\_p)}\right)$$

$$\frac{d\left([PDL1\_mRNA]\cdot V_{\text{"Neuroblastoma Cell Nucleus"}}\right)}{dt} = +V_{\text{"Neuroblastoma Cell Nucleus"}}\cdot \text{"Henri-Michaelis-Menten (irreversible)"}\left([STAT3\_ac\_p], Km_{(PDL1\_transcription\_by\_STAT3\_ac\_p)}, V_{(PDL1\_transcription\_by\_STAT3\_ac\_p)}\right)$$

$$+V_{\text{"Neuroblastoma Cell Nucleus"}}\cdot \text{"Henri-Michaelis-Menten (irreversible)"}\left([STAT1\_p], Km_{(PDL1\_transcription\_by\_STAT1\_p)}, V_{(PDL1\_transcription\_by\_STAT1\_p)}\right)$$

$$-V_{\text{"Neuroblastoma Cell Nucleus"}}\cdot k1_{(\text{"PDL1\_mRNA degradation"})}\cdot [PDL1\_mRNA]$$

$$+V_{\text{"Neuroblastoma Cell Nucleus"}}\cdot \text{"Henri-Michaelis-Menten (irreversible)"}\left([STAT3\_p], Km_{(PDL1\_transcription\_by\_STAT3\_p)}, V_{(PDL1\_transcription\_by\_STAT3\_p)}\right)$$

$$\frac{d\left([degraded\_PDL1\_mRNA]\cdot V_{\text{"Neuroblastoma Cell Nucleus"}}\right)}{dt} = +V_{\text{"Neuroblastoma Cell Nucleus"}}\cdot k1_{(\text{"PDL1\_mRNA degradation"})}\cdot [PDL1\_mRNA]$$

$$\frac{d\left([ASK/MLK\_active]\cdot V_{\text{"Neuroblastoma Cell Cytoplasm"}}\right)}{dt} = +V_{\text{"Neuroblastoma Cell Cytoplasm"}}\cdot \text{HMM\_Modified}\left(Kcat_{(ASK/MLK\_activation\_by\_JNK)}, km_{(ASK/MLK\_activation\_by\_JNK)}, [JNK\_active], [ASK/MLK\_inactive]\right)$$

$$-V_{\text{"Neuroblastoma Cell Cytoplasm"}}\cdot \text{"HMM modified"}\left(Kcat_{(ASK/MLK\_inhibition\_by\_Akt)}, [Akt\_active], [ASK/MLK\_active], km_{(ASK/MLK\_inhibition\_by\_Akt)}\right)$$

$$\frac{d\left([ASK/MLK\_inactive]\cdot V_{\text{"Neuroblastoma Cell Cytoplasm"}}\right)}{dt} = -V_{\text{"Neuroblastoma Cell Cytoplasm"}}\cdot \text{HMM\_Modified}\left(Kcat_{(ASK/MLK\_activation\_by\_JNK)}, km_{(ASK/MLK\_activation\_by\_JNK)}, [JNK\_active], [ASK/MLK\_inactive]\right)$$

$$+V_{\text{"Neuroblastoma Cell Cytoplasm"}}\cdot \text{"HMM modified"}\left(Kcat_{(ASK/MLK\_inhibition\_by\_Akt)}, [Akt\_active], [ASK/MLK\_active], km_{(ASK/MLK\_inhibition\_by\_Akt)}\right)$$

	Name	Compartment	Type	Unit	Initial Concentration [Unit]
1	EGFR_free	Neuroblastoma Cell Cytoplasm	reactions	mmol/ml	618
2	EGFR_bound	Neuroblastoma Cell Cytoplasm	reactions	mmol/ml	0
3	SOS_active	Neuroblastoma Cell Cytoplasm	reactions	mmol/ml	0
4	SOS_inactive	Neuroblastoma Cell Cytoplasm	reactions	mmol/ml	9456
5	EGF	Extracellular Space	fixed	mmol/ml	10000000
6	NGF	Extracellular Space	fixed	mmol/ml	10000000
7	NGFR_bound	Neuroblastoma Cell Cytoplasm	reactions	mmol/ml	0
8	NGFR	Neuroblastoma Cell Cytoplasm	reactions	mmol/ml	14737
9	P90Rsk_active	Neuroblastoma Cell Cytoplasm	reactions	mmol/ml	0
10	P90Rsk_inactive	Neuroblastoma Cell Cytoplasm	reactions	mmol/ml	30899
11	RAS_active	Neuroblastoma Cell Cytoplasm	reactions	mmol/ml	0
12	RAS_inactive	Neuroblastoma Cell Cytoplasm	reactions	mmol/ml	12153
13	RasGap_active	Neuroblastoma Cell Cytoplasm	fixed	mmol/ml	10143
14	PTEN_active	Neuroblastoma Cell Cytoplasm	reactions	mmol/ml	0
15	TCL1	Neuroblastoma Cell Cytoplasm	fixed	mmol/ml	123
16	CTMP	Neuroblastoma Cell Cytoplasm	fixed	mmol/ml	9570
17	S6K1_inactive	Neuroblastoma Cell Cytoplasm	reactions	mmol/ml	9790
18	mTORC1_inactive	Neuroblastoma Cell Cytoplasm	reactions	mmol/ml	13351
19	Rap1Gap	Neuroblastoma Cell Cytoplasm	fixed	mmol/ml	5334
20	HSP90-Cdc37Active	Neuroblastoma Cell Cytoplasm	fixed	mmol/ml	79790
21	PDK1_inactive	Neuroblastoma Cell Cytoplasm	reactions	mmol/ml	3120
22	IRS1_inactive	Neuroblastoma Cell Cytoplasm	reactions	mmol/ml	1922
23	PDK1_active	Neuroblastoma Cell Cytoplasm	reactions	mmol/ml	0
24	PHLPP	Neuroblastoma Cell Cytoplasm	fixed	mmol/ml	2584
25	mTORC1_active	Neuroblastoma Cell Cytoplasm	reactions	mmol/ml	0

26	S6K1_active	Neuroblastoma Cell Cytoplasm	reactions	mmol/ml	0
27	mTORC2_active	Neuroblastoma Cell Cytoplasm	fixed	mmol/ml	3685
28	IRS1_active	Neuroblastoma Cell Cytoplasm	reactions	mmol/ml	0
29	Akt_inactive	Neuroblastoma Cell Cytoplasm	reactions	mmol/ml	0
30	C3G_inactive	Neuroblastoma Cell Cytoplasm	reactions	mmol/ml	15703
31	Raf1_inactive	Neuroblastoma Cell Cytoplasm	reactions	mmol/ml	34479
32	degradedEGFR	Neuroblastoma Cell Cytoplasm	reactions	mmol/ml	0
33	Akt_active	Neuroblastoma Cell Cytoplasm	reactions	mmol/ml	48554
34	C3G_active	Neuroblastoma Cell Cytoplasm	reactions	mmol/ml	0
35	Rap1_active	Neuroblastoma Cell Cytoplasm	reactions	mmol/ml	0
36	Rap1_inactive	Neuroblastoma Cell Cytoplasm	reactions	mmol/ml	15629
37	bRaf_active	Neuroblastoma Cell Cytoplasm	reactions	mmol/ml	0
38	PP2A_active	Neuroblastoma Cell Cytoplasm	reactions	mmol/ml	0
39	Mek_active	Neuroblastoma Cell Cytoplasm	reactions	mmol/ml	0
40	Raf1PPtase	Neuroblastoma Cell Cytoplasm	fixed	mmol/ml	47458
41	bRaf_inactive	Neuroblastoma Cell Cytoplasm	reactions	mmol/ml	4857
42	ERK_inactive	Neuroblastoma Cell Cytoplasm	reactions	mmol/ml	31120
43	PI3K_active	Neuroblastoma Cell Cytoplasm	reactions	mmol/ml	0
44	PI3K_inactive	Neuroblastoma Cell Cytoplasm	reactions	mmol/ml	3657
45	ERK_active	Neuroblastoma Cell Cytoplasm	reactions	mmol/ml	0
46	Raf1_active	Neuroblastoma Cell Cytoplasm	reactions	mmol/ml	0
47	Mek_inactive	Neuroblastoma Cell Cytoplasm	reactions	mmol/ml	33361
48	degradedNGFR	Neuroblastoma Cell Cytoplasm	reactions	mmol/ml	0
49	RHEB	Neuroblastoma Cell Cytoplasm	fixed	mmol/ml	21021
50	STAT1	Neuroblastoma Cell Nucleus	reactions	mmol/ml	17898
51	STAT1_p	Neuroblastoma Cell Nucleus	reactions	mmol/ml	0
52	STAT3	Neuroblastoma Cell	reactions	mmol/ml	30043

		Nucleus			
53	STAT3_ac	Neuroblastoma Cell Nucleus	reactions	mmol/ml	0
54	CREBBP	Neuroblastoma Cell Nucleus	fixed	mmol/ml	9715
55	STAT3_ac_p	Neuroblastoma Cell Nucleus	reactions	mmol/ml	0
56	PIAS3	Neuroblastoma Cell Nucleus	fixed	mmol/ml	10445
57	STAT3_p	Neuroblastoma Cell Nucleus	reactions	mmol/ml	0
58	PDL1_mRNA	Neuroblastoma Cell Nucleus	reactions	mmol/ml	0
59	degraded_PDL1_mRNA	Neuroblastoma Cell Nucleus	reactions	mmol/ml	0
60	ASK/MLK_active	Neuroblastoma Cell Cytoplasm	reactions	mmol/ml	1
61	ASK/MLK_inactive	Neuroblastoma Cell Cytoplasm	reactions	mmol/ml	11079
62	JNK_active	Neuroblastoma Cell Cytoplasm	reactions	mmol/ml	1
63	MKK3/6_active	Neuroblastoma Cell Cytoplasm	reactions	mmol/ml	1
64	MKK3/6_inactive	Neuroblastoma Cell Cytoplasm	reactions	mmol/ml	2235
65	p38_active	Neuroblastoma Cell Nucleus	reactions	mmol/ml	0
66	p38_inactive	Neuroblastoma Cell Nucleus	reactions	mmol/ml	25347
67	PP2A_inactive	Neuroblastoma Cell Cytoplasm	reactions	mmol/ml	15532
68	DUSP4/6_active	Neuroblastoma Cell Nucleus	reactions	mmol/ml	0
69	DUSP4/6_inactive	Neuroblastoma Cell Nucleus	reactions	mmol/ml	2764
70	JNK_inactive	Neuroblastoma Cell Cytoplasm	reactions	mmol/ml	18643
71	MKK4/7_active	Neuroblastoma Cell Cytoplasm	reactions	mmol/ml	1
72	MKK4/7_inactive	Neuroblastoma Cell Cytoplasm	reactions	mmol/ml	11269
73	DUSP2_active	Neuroblastoma Cell Nucleus	reactions	mmol/ml	0
74	DUSP2_inactive	Neuroblastoma Cell Nucleus	reactions	mmol/ml	2334
75	MYCN_mRNA	Neuroblastoma Cell Nucleus	reactions	mmol/ml	0
76	GSK3b_active	Neuroblastoma Cell Cytoplasm	reactions	mmol/ml	0
77	GSK3b_inactive	Neuroblastoma Cell Cytoplasm	reactions	mmol/ml	11711
78	N-MYC	Neuroblastoma Cell Cytoplasm	reactions	mmol/ml	0

79	MYCN	Neuroblastoma Cell Cytoplasm	fixed	mmol/ml	17819
80	MYCN_mRNA_degraded	Neuroblastoma Cell Nucleus	reactions	mmol/ml	0
81	N-MYC_pp	Neuroblastoma Cell Cytoplasm	reactions	mmol/ml	0
82	ALK_Mutated	Neuroblastoma Cell Cytoplasm	reactions	mmol/ml	8510
83	Akt_Thr308	Neuroblastoma Cell Cytoplasm	reactions	mmol/ml	1
84	Akt_conformational_change	Neuroblastoma Cell Cytoplasm	reactions	mmol/ml	1
85	PIP3	Neuroblastoma Cell Cytoplasm	reactions	mmol/ml	100000
86	PIP2	Neuroblastoma Cell Cytoplasm	reactions	mmol/ml	0
87	SOCS1	Neuroblastoma Cell Cytoplasm	fixed	mmol/ml	1281
88	SOCS3	Neuroblastoma Cell Cytoplasm	fixed	mmol/ml	10186
89	AMPK_active	Neuroblastoma Cell Cytoplasm	reactions	mmol/ml	0
90	AMPK_inactive	Neuroblastoma Cell Cytoplasm	reactions	mmol/ml	1330
91	PTEN_inactive	Neuroblastoma Cell Cytoplasm	reactions	mmol/ml	10130
92	proNGFR	Neuroblastoma Cell Cytoplasm	fixed	mmol/ml	14737
93	Crizotinib	Neuroblastoma Cell Cytoplasm	reactions	mmol/ml	1,40E-06
94	EGFR_inhibited	Neuroblastoma Cell Cytoplasm	reactions	mmol/ml	0
95	Gefitinib	Neuroblastoma Cell Cytoplasm	reactions	mmol/ml	3,00E-06

Colonna1	Name	Reaction	Rate Law	Km	Kcat	k1	k2	k3	k4	k5	References
1	Biding EGF with EGFR	EGF + EGFR_free = EGFR_bound; EGF	Mass action (reversible)			2,18503E-05	0,121008				[1]
2	SOS_activation_by_EGFR_bound	SOS_inactive -> SOS_active; EGFR_bound	SOS activation by EGFR	6086070	694.731						[1]
3	SOS activation by NGF	SOS_inactive -> SOS_active; NGFR_bound	SOS activation by NGF	2112,66	389.428						[2]
4	Biding NGF	NGF + NGFR = NGFR_bound ; NGF	Mass action (reversible)			1,38E-07	0,0072				[2]
5	SOS deactivation by P90Rsk	SOS_active -> SOS_inactive; P90Rsk_active	SOS deactivation by P90Rsk			360.000	647.019				[1]
6	SOS deactivation by ERK	SOS_active -> SOS_inactive; ERK_active	SOS deactivation by ERK	763523	0,0213697						[1]
7	P90Rsk Activation	P90Rsk_inactive -> P90Rsk_active; ERK_active	P90Rsk Activation	360000	0,0100758						[1]
8	RAS Activation	RAS_inactive -> RAS_active; SOS_active	RAS Activation	35954,3	32.344						[1]
9	RAS deactivation	RAS_active -> RAS_inactive;	HMM modified	73733,3	776.942						[1]

		RasGap_active					
10	Raf1_Feedback_Deactivation_Raf1PPtase	Raf1_active -> Raf1_inactive ; Raf1PPtase	HMM_Modified	1061,71	0,126329		3
11	Mek_Activation_Raf1	Mek_inactive -> Mek_active; Raf1_active	HMM_Modified	1800000	70.122		3
12	Mek_Feedback_Deactivation_PP2A	Mek_active -> Mek_inactive ; PP2A_active	HMM_Modified	518753	283.243		1
13	Erk_Activation	ERK_inactive -> ERK_active; Mek_active	HMM_Modified	782264	7.652		4
14	Erk_Feedback_Deactivation_PP2A	ERK_active -> ERK_inactive; PP2A_active	HMM_Modified	1630000	414.072		5
15	C3G_Deactivation	C3G_active -> C3G_inactive	Mass action (irreversible)			2,5	1
16	Mek_Activation_bRaf	Mek_inactive -> Mek_active; bRaf_active	HMM_Modified	4768350	185.759		1
17	PIP3_Activation	PIP2 -> PIP3; PI3K_active	HMM_Modified	653951	0,0566279		1
18	bRaf_Deactivation_Raf1PPtase	bRaf_active -> bRaf_inactive ; Raf1PPtase	HMM_Modified	2052,32	0,0832448		2
19	PIP3_Feedba	PIP3 -> PIP2;	HMM_Modifi	518753	283.243		1

	ck_Deactivation_PTEN	PTEN_active	ed					
20	PI3K_Deactivation	PI3K_active -> PI3K_inactive	Mass action (irreversible)				0,005	1
21	Rap1_Activation	Rap1_inactive -> Rap1_active; C3G_active	HMM_Modified	10965,6	140.145			6
22	bRaf_Activation_Rap1	bRaf_inactive -> bRaf_active; Rap1_active	HMM_Modified	360000	0,776			1
23	Raf1_Deactivation_by_Akt	Raf1_active -> Raf1_inactive; Akt_active	HMM_Modified	15781,3	199.935			3
24	Rap1_Feedback_Deactivation_Rap1Gap	Rap1_active -> Rap1_inactive; Rap1Gap	HMM_Modified	43859,1	404.006			7
25	Akt_Activation_PIP3	Akt_inactive -> Akt_conformational_change; PIP3	HMM_Modified	653951	0,0566279			1
26	PI3K_Activation_IRS1	PI3K_inactive -> PI3K_active; IRS1_active	HMM_Modified	272056	0,0771067			1
27	Akt_Deactivation_PP2A	Akt_active -> Akt_inactive; PP2A_active	HMM_Modified	2476,85	0,296393			8
28	PDK1_Activation	PDK1_inactive ->	HMM_Modified	1007340	985.367			9

		PDK1_active; PIP3					
29	Akt_Deactivation_PHLPP	Akt_active -> Akt_inactive; PHLPP	HMM_Modified	24761,9	1423,62		10
30	Akt_Deactivation_CTMP	Akt_active -> Akt_inactive; CTMP	HMM_Modified	15670,1	0,0409454		11
31	PDK1_Deactivation	PDK1_active -> PDK1_inactive	Mass action (irreversible)			2,5	1
32	Akt_Activation_mTORC2	Akt_Thr308 -> Akt_active; mTORC2_active	HMM_Modified	149.078	0,00999962		12
33	S6K1_Activation_mTORC1	S6K1_inactive -> S6K1_active; mTORC1_active	HMM_Modified	763523	0,0213697		1
34	Akt_Activation_HSP90-Cdc37	Akt_inactive -> Akt_active; HSP90-Cdc37Active	HMM_Modified	653951	0,0566279		10
35	Akt_Activation_PDK1	Akt_conformational_change -> Akt_Thr308; PDK1_active	HMM_Modified	10335,5	0,00000902054		9
36	Akt_Activation_TCL1	Akt_inactive -> Akt_active;	HMM_Modified	0,337626	19909,2		10

		TCL1						
37	Erk_Feedback_Deactivation_Raf1	ERK_active -> ERK_inactive; Raf1_active	HMM_Modified	3496490	88.912			[1]
38	mTORC1_Activation_RHEB	mTORC1_inactive -> mTORC1_active; RHEB	HMM_Modified	119355	151.212			[13]
39	IRS1_Feedback_Deactivation_S6K1	IRS1_active -> IRS1_inactive; S6K1_active	HMM_Modified	896896	1611,97			[1]
40	EGFR_free degradation	EGFR_free -> degradedEGFR	Mass action (irreversible)				0,00125	[1]
41	NGFR degradation	NGFR -> degradedNGFR	Mass action (irreversible)				0,2	[2]
42	bNGFR degradation	NGFR_bound -> degradedNGFR	Mass action (irreversible)				0,2	[2]
43	Raf1 activation_by_Ras	Raf1_inactive -> Raf1_active; RAS_active	HMM_Modified	1432410	1509,36			[3]
44	Raf1_deactivation_by_ERK	Raf1_active -> Raf1_inactive; ERK_active	HMM_Modified	68301,1	0,003928			[3]
45	PI3K_Activation_Ras	PI3K_inactive -> PI3K_active; RAS_active	HMM_Modified	272056	0,0771067			[1]
46	PI3K_Activati	PI3K_inactive	HMM_Modifi	184912	106.737			[1]

	on_EGFR_di_p	-> PI3K_active; EGFR_bound	ed								
47	C3G_Activation_NGFR	C3G_inactive -> C3G_active; NGFR_bound	HMM_Modified	12876,2	146.912						[2]
48	P90Rsk_deactivation	P90Rsk_active -> P90Rsk_inactive	Mass action (irreversible)				0,005				[1]
49	bRAF_activation_RAS	bRaf_inactive -> bRaf_active; RAS_active	HMM_Modified	360000	0,776						[2]
50	IRS1_Activation	IRS1_inactive -> IRS1_active; EGFR_bound	HMM_Modified	184912	106.737						[1]
51	STAT1_activation	STAT1 -> STAT1_p; EGFR_bound PIAS3 SOCS1 SOCS3	HMM_activation and inhibition_STAT1	1	0,419	0,3	0,3	19	0,1		[14]
52	STAT3_acetilation	STAT3 -> STAT3_ac; CREBBP	HMM_Modified	0,1	37,7						[14]
53	STAT3_phosphorilation_by_EGFR_bound	STAT3_ac -> STAT3_ac_p; EGFR_bound PIAS3 SOCS1 SOCS3 STAT3_p	HMM_activation and inhibition_STAT3	1	0,419	0,3	0,3	19	0,1	0,00062	[14]
54	STAT3_phosporilation_by_	STAT3 -> STAT3_p;	HMM modified	1467,82	203.596						[15]

	mTORC1	mTORC1_active						
55	PDL1_transcription_by_STAT3_ac_p	STAT3_ac_p -> PDL1_mRNA	Henri-Michaelis-Menten (irreversible)	0,918449	0,0301143			16
56	PDL1_transcription_by_STAT1_p	STAT1_p -> PDL1_mRNA	Henri-Michaelis-Menten (irreversible)	0,00549287	154.143			17
57	PDL1_mRNA degradation	PDL1_mRNA -> degraded_PDL1_mRNA	Mass action (irreversible)				0,0000001	16
58	ASK/MLK_activation_by_JNK	ASK/MLK_inactive -> ASK/MLK_active; JNK_active	HMM_Modified	193.443	677.718			18
59	MKK3/6_activation	MKK3/6_inactive -> MKK3/6_active	Henri-Michaelis-Menten (irreversible)	0,0889526	323.773			19
60	p38_activation	p38_inactive -> p38_active; MKK3/6_active	HMM_Modified	0,0288693	1751,2			20
61	PP2A_activation_by_p38	PP2A_inactive -> PP2A_active; p38_active	HMM_Modified	60421,9	1,14398E-05			20
62	DUSP4/6_activation_by_ERK	DUSP4/6_inactive -> DUSP4/6_acti	HMM_Modified	3,35444E-05	9,15995E-05			21

		ve; ERK_active					
63	JNK_inhibition_by_DUSP4/6	JNK_active -> JNK_inactive; DUSP4/6_active	HMM_Modified	9,03661E-06	13732		[22]
64	MKK4/7_activation	MKK4/7_inactive -> MKK4/7_active; ASK/MLK_active	HMM_Modified	0,000001	0,590759		[23]
65	JNK_activation_by_MKK4/7	JNK_inactive -> JNK_active; MKK4/7_active	HMM_Modified	0,000001	16345,5		[24]
66	MKK4/7_inhibition_by_Akt	MKK4/7_active -> MKK4/7_inactive; Akt_active	HMM_Modified	0,466352	0,000188165		[25]
67	DUSP2_transcription_by_JNK	DUSP2_inactive -> DUSP2_active ; JNK_active	HMM_Modified	0,30431	2068,53		[26]
68	ERK_inhibition_by_DUSP2	ERK_active -> ERK_inactive; DUSP2_active	HMM_Modified	707382	0,246066		[26]
69	p38_inhibition_by_DUSP2	p38_active -> p38_inactive; DUSP2_active	HMM_Modified	0,00181113	2,0056E-06		[26]
70	N-MYC_degradation_by_GS	N-MYC -> N-MYC_pp; GSK3b_active	HMM_Modified	2,32369E-06	0,4767		[27]

	K3b							
71	GSK3b_inactivation_by_Akt	GSK3b_active -> GSK3b_inactive; Akt_active	HMM_Modified	6291,13	0,445559			28
72	MYCN_transcription_by_STAT3_p	MYCN -> MYCN_mRNA ; STAT3_p	HMM_Modified	625.826	4358,93			29
73	MYCN_mRNA_degradation	MYCN_mRNA -> MYCN_mRNA_degraded	Mass action (irreversible)				0,00177055	30
74	DUSP2_transcription_by_N-MYC	DUSP2_inactive -> DUSP2_active ; N-MYC	HMM modified	0,000713062	0,00372918			26
75	PI3K_activation_by_ALK	PI3K_inactive -> PI3K_active; ALK_active	HMM_Modified	6827,58	0,225075			31
76	STAT3_phosphorilation_by_ALK	STAT3 -> STAT3_p; ALK_active	HMM_Modified	155.479	0,136315			32
77	EGFR_bound_degradation	EGFR_bound -> degradedEGFR	Mass action (irreversible)				0,1	2
78	PDL1_transcription_by_STAT3_p	STAT3_p -> PDL1_mRNA	Henri-Michaelis-Menten (irreversible)	0,918449	0,0301143			16
79	ALK_activation	ALK_inactive = ALK_active	Mass action (reversible)			485.982	0,00328375	32
80	AMPK_activa	AMPK_inactiv	Mass action			0,959653	0,912883	33

	tion	e = AMPK_active	(reversible)							
81	mTORC1_inhibition_by_AMPK	mTORC1_active -> mTORC1_inactive; AMPK_active	HMM_Modified	0,0011625	0,0487685					[33]
82	PTEN_activation	PTEN_inactive = PTEN_active	Mass action (reversible)			6,62893E-05	0,00146868			[34]
83	ASK/MLK_inhibition_by_Akt	ASK/MLK_active -> ASK/MLK_inactive; Akt_active	HMM modified	815.976	0,000001					[18]
84	N-MYC_translation_by_MYCN_mRNA	MYCN_mRNA -> N-MYC	Henri-Michaelis-Menten (irreversible)	625.826	4358,93					[27]
85	ERK_activation_by_ALK	ERK_inactive -> ERK_active; ALK_active	HMM modified	2073,56	0,000952579					[31]
86	Crizotinib	ALK_Mutated -> ; Crizotinib	HMM modified	0,00319548	6,82E+12					[35]
87	Crizotinib Degradation	Crizotinib ->	Mass action (irreversible)				5,92527E-08			[35]
88	EGFR_inhibitor	EGFR_free -> EGFR_inhibited; Gefitinib	HMM modified	0,025911357	524895778,4					[36]
89	Gefitinib Degradation	Gefitinib ->	Mass action (irreversible)				2,92527E-07			[36]

1. Pappalardo, F. *et al.* Computational modeling of PI3K/AKT and MAPK signaling pathways in melanoma cancer. *PLoS One* (2016).

doi:10.1371/journal.pone.0152104

2. Brown, K. S. *et al.* The statistical mechanics of complex signaling networks: Nerve growth factor signaling. *Phys. Biol.* (2004). doi:10.1088/1478-3967/1/3/006

3. Rushworth, L. K., Hindley, A. D., O'Neill, E. & Kolch, W. Regulation and Role of Raf-1/B-Raf Heterodimerization. *Mol. Cell. Biol.* (2006). doi:10.1128/MCB.26.6.2262-2272.2006

4. Cruz, C. & Cruz, F. The ERK 1 and 2 Pathway in the Nervous System: From Basic Aspects to Possible Clinical Applications in Pain and Visceral Dysfunction. *Curr. Neuropharmacol.* (2007). doi:10.2174/157015907782793630

5. Yu, L. G., Packman, L. C., Weldon, M., Hamlett, J. & Rhodes, J. M. Protein phosphatase 2A, a negative regulator of the ERK signaling pathway, is activated by tyrosine phosphorylation of putative HLA class II-associated protein I (PHAPI)/pp32 in response to the antiproliferative lectin, jacalin. *J. Biol. Chem.* (2004). doi:10.1074/jbc.M400017200

6. Hattori, M. & Minato, N. Rap1 GTPase: Functions, Regulation, and Malignancy. *Journal of Biochemistry* (2003). doi:10.1093/jb/mvg180

7. Kupzig, S., Bouyoucef-Cherchalli, D., Yarwood, S., Sessions, R. & Cullen, P. J. The Ability of GAP1IP4BP To Function as a Rap1 GTPase-Activating Protein (GAP) Requires Its Ras GAP-Related Domain and an Arginine Finger Rather than an Asparagine Thumb. *Mol. Cell. Biol.* (2009). doi:10.1128/MCB.00427-09

8. Vadlakonda, L., Pasupuleti, M. & Pallu, R. Role of PI3K-AKT-mTOR and Wnt Signaling Pathways in Transition of G1-S Phase of Cell Cycle in Cancer Cells. *Front. Oncol.* (2013). doi:10.1088/1361-6463/aa6fbb

9. Ju, R. & Simons, M. Syndecan 4 regulation of PDK1-dependent Akt activation. *Cell. Signal.* (2013). doi:10.1016/j.cellsig.2012.09.007

10. Pekarsky, Y. *et al.* Tcl1 enhances Akt kinase activity and mediates its nuclear translocation. *Proc. Natl. Acad. Sci. U. S. A.* (2000). doi:10.1073/pnas.040557697

11. Nicholson, K. M. & Anderson, N. G. The protein kinase B/Akt signalling pathway in human malignancy. *Cellular Signalling* (2002). doi:10.1016/S0898-6568(01)00271-6

12. Yang, G., Murashige, D. S., Humphrey, S. J. & James, D. E. A Positive Feedback Loop between Akt and mTORC2 via SIN1 Phosphorylation. *Cell Rep.* (2015). doi:10.1016/j.celrep.2015.07.016

13. Sato, T., Umetsu, A. & Tamanoi, F. Characterization of the Rheb-mTOR Signaling Pathway in Mammalian Cells: Constitutive Active Mutants of Rheb and mTOR. *Methods in Enzymology* (2008). doi:10.1016/S0076-6879(07)38021-X

14. Brogden, K. A. *et al.* Genomics of NSCLC patients both affirm PD-L1 expression and predict their clinical responses to anti-PD-1 immunotherapy. *BMC Cancer* **18,** (2018).

15. Yokogami, K., Wakisaka, S., Avruch, J. & Reeves, S. A. Serine phosphorylation and maximal activation of STAT3 during CNTF signaling is mediated by the rapamycin target mTOR. *Curr. Biol.* (2000). doi:10.1016/S0960-9822(99)00268-7

16. Wolfle, S. J. *et al.* PD-L1 expression on tolerogenic APCs is controlled by STAT-3. *Eur. J. Immunol.* (2011). doi:10.1002/eji.201040979

17. Moon, J. W. *et al.* IFNγ induces PD-L1 overexpression by JAK2/STAT1/IRF-1 signaling in EBV-positive gastric carcinoma. *Sci. Rep.* (2017). doi:10.1038/s41598-017-18132-0

18. Shen, K. *et al.* Cambogin Induces Caspase-Independent Apoptosis through the ROS/JNK Pathway and Epigenetic Regulation in Breast Cancer Cells. *Mol. Cancer Ther.* (2015). doi:10.1158/1535-7163.MCT-14-1048

19. Wang, W. *et al.* Sequential Activation of the MEK-Extracellular Signal-Regulated Kinase and MKK3/6-p38 Mitogen-Activated Protein Kinase Pathways Mediates Oncogenic ras-Induced Premature Senescence. *Mol. Cell. Biol.* (2002). doi:10.1128/MCB.22.10.3389-3403.2002

20. Staples, C. J., Owens, D. M., Maier, J. V., Cato, A. C. B. & Keyse, S. M. Cross-talk between the p38α and JNK MAPK pathways mediated by MAP kinase phosphatase-1 determines cellular sensitivity to UV radiation. *J. Biol. Chem.* (2010). doi:10.1074/jbc.M110.117911

21. Cagnol, S. & Rivard, N. Oncogenic KRAS and BRAF activation of the MEK/ERK signaling pathway promotes expression of dual-specificity phosphatase 4 (DUSP4/MKP2) resulting in nuclear ERK1/2 inhibition. *Oncogene* (2013). doi:10.1038/onc.2012.88

22. Schmid, C. A. *et al.* DUSP4 deficiency caused by promoter hypermethylation drives JNK signaling and tumor cell survival in diffuse large B cell lymphoma. *J. Exp. Med.* (2015). doi:10.1084/jem.20141957

23. Tournier, C. *et al.* MKK7 is an essential component of the JNK signal transduction pathway activated by proinflammatory cytokines. *Genes Dev.* (2001). doi:10.1101/gad.888501

24. Wang, R. *et al.* Inhibition of MLK3-MKK4/7-JNK1/2 pathway by Akt1 in exogenous estrogen-induced neuroprotection against transient global cerebral ischemia by a non-genomic mechanism in male rats. *J. Neurochem.* (2006). doi:10.1111/j.1471-4159.2006.04201.x

25. Martelli, A. M., Evangelisti, C., Chiarini, F., Grimaldi, C. & McCubrey, J. A. The emerging role of the phosphatidylinositol 3-Kinase/ AKt/Mammalian target of rapamycin signaling network in cancer stem cell biology. *Cancers* (2010). doi:10.3390/cancers2031576

26. Chappell, J., Sun, Y., Singh, A. & Dalton, S. MYC/MAX control ERK signaling and pluripotency by regulation of dual-specificity phosphatases 2 and 7. *Genes Dev.* (2013). doi:10.1101/gad.211300.112

27. Vaughan, L. *et al.* Inhibition of mTOR-kinase destabilizes MYCN and is a potential therapy for MYCN-dependent tumors. *Oncotarget* **7,** 57525–57544 (2016).

28. Wakatsuki, S., Saitoh, F. & Araki, T. ZNRF1 promotes Wallerian degeneration by degrading AKT to induce GSK3B-dependent CRMP2 phosphorylation. *Nat. Cell Biol.* (2011). doi:10.1038/ncb2373

29. Hadjidaniel, M. D. *et al.* Tumor-associated macrophages promote neuroblastoma via STAT3 phosphorylation and up-regulation of c-MYC. *Oncotarget* (2017). doi:10.18632/oncotarget.21066

30. Gu, L. *et al.* MDM2 regulates MYCN mRNA stabilization and translation in human neuroblastoma cells. *Oncogene* (2012). doi:10.1038/onc.2011.343

31. Tucker, E. R. *et al.* Immunoassays for the quantification of ALK and phosphorylated ALK support the evaluation of on-target ALK inhibitors in neuroblastoma. *Mol. Oncol.* (2017). doi:10.1002/1878-0261.12069

32. Sattu, K. *et al.* Phosphoproteomic analysis of anaplastic lymphoma kinase (ALK) downstream signaling pathways identifies signal transducer and activator of transcription 3 as a functional target of activated ALK in neuroblastoma cells. in *FEBS Journal* (2013). doi:10.1111/febs.12453

33. Gwinn, D. M. *et al.* AMPK Phosphorylation of Raptor Mediates a Metabolic Checkpoint. *Mol. Cell* (2008). doi:10.1016/j.molcel.2008.03.003

34. Moritake, H., Horii, Y., Kuroda, H. & Sugimoto, T. Analysis of PTEN/MMAC1 alteration in neuroblastoma. *Cancer Genet. Cytogenet.* (2001). doi:10.1016/S0165-4608(00)00378-2

35. Kogita, A. *et al.* Activated MET acts as a salvage signal after treatment with alectinib, a selective ALK inhibitor, in ALK-positive non-small cell lung cancer. *Int. J. Oncol.* (2015). doi:10.3892/ijo.2014.2797

36. Costa, D. B. *et al.* BIM mediates EGFR tyrosine kinase inhibitor-induced apoptosis in lung cancers with oncogenic EGFR mutations. *PLoS Med.* (2007). doi:10.1371/journal.pmed.0040315
